# Land‐based measures to mitigate climate change: Potential and feasibility by country

**DOI:** 10.1111/gcb.15873

**Published:** 2021-10-11

**Authors:** Stephanie Roe, Charlotte Streck, Robert Beach, Jonah Busch, Melissa Chapman, Vassilis Daioglou, Andre Deppermann, Jonathan Doelman, Jeremy Emmet‐Booth, Jens Engelmann, Oliver Fricko, Chad Frischmann, Jason Funk, Giacomo Grassi, Bronson Griscom, Petr Havlik, Steef Hanssen, Florian Humpenöder, David Landholm, Guy Lomax, Johannes Lehmann, Leah Mesnildrey, Gert‐Jan Nabuurs, Alexander Popp, Charlotte Rivard, Jonathan Sanderman, Brent Sohngen, Pete Smith, Elke Stehfest, Dominic Woolf, Deborah Lawrence

**Affiliations:** ^1^ Department of Environmental Sciences University of Virginia Charlottesville Virginia USA; ^2^ Climate Focus Berlin Germany; ^3^ International Politics University of Potsdam Potsdam Germany; ^4^ Environmental Engineering and Economics Division RTI International Research Triangle Park North Carolina USA; ^5^ Conservation International Arlington Virginia USA; ^6^ Department of Environmental Science, Policy, and Management University of California Berkeley Berkeley California USA; ^7^ Copernicus Institute of Sustainable Development Utrecht University Utrecht the Netherlands; ^8^ PBL Netherlands Environmental Assessment Agency The Hague the Netherlands; ^9^ International Institute for Applied Systems Analysis (IIASA) Laxenburg Austria; ^10^ New Zealand Agricultural Greenhouse Gas Research Centre Palmerston North New Zealand; ^11^ Department of Agricultural and Applied Economics University of Wisconsin‐Madison Madison Wisconsin USA; ^12^ Project Drawdown San Francisco California USA; ^13^ Land Use and Climate Knowledge Initiative Chicago Illinois USA; ^14^ Joint Research Centre European Commission Ispra Italy; ^15^ Department of Environmental Science Radboud University Nijmegen Nijmegen The Netherlands; ^16^ Potsdam Institute for Climate Impact Research (PIK), Member of the Leibniz Association Potsdam Germany; ^17^ College of Engineering, Mathematics and Physical Sciences University of Exeter Exeter UK; ^18^ Soil and Crop Science School of Integrative Plant Science College of Agriculture and Life Science Cornell University Ithaca New York USA; ^19^ Sciences Po Paris Paris School of International Affairs (PSIA) Paris France; ^20^ Wageningen Environmental Research Wageningen University and Research Wageningen the Netherlands; ^21^ Forest Ecology and Forest Management Group Wageningen University Wageningen the Netherlands; ^22^ Woodwell Climate Research Center Falmouth Massachusetts USA; ^23^ Department of Agricultural, Environmental and Development Economics Ohio State University Columbus Ohio USA; ^24^ Institute of Biological and Environmental Sciences University of Aberdeen Aberdeen UK

**Keywords:** AFOLU, co‐benefits, demand management, feasibility, land management, land sector, mitigation, natural climate solutions, nature‐based solutions

## Abstract

Land‐based climate mitigation measures have gained significant attention and importance in public and private sector climate policies. Building on previous studies, we refine and update the mitigation potentials for 20 land‐based measures in >200 countries and five regions, comparing “bottom‐up” sectoral estimates with integrated assessment models (IAMs). We also assess implementation feasibility at the country level. Cost‐effective (available up to $100/tCO_2_eq) land‐based mitigation is 8–13.8 GtCO_2_eq yr^−1^ between 2020 and 2050, with the bottom end of this range representing the IAM median and the upper end representing the sectoral estimate. The cost‐effective sectoral estimate is about 40% of available technical potential and is in line with achieving a 1.5°C pathway in 2050. Compared to technical potentials, cost‐effective estimates represent a more realistic and actionable target for policy. The cost‐effective potential is approximately 50% from forests and other ecosystems, 35% from agriculture, and 15% from demand‐side measures. The potential varies sixfold across the five regions assessed (0.75–4.8 GtCO2eq yr^−1^) and the top 15 countries account for about 60% of the global potential. Protection of forests and other ecosystems and demand‐side measures present particularly high mitigation efficiency, high provision of co‐benefits, and relatively lower costs. The feasibility assessment suggests that governance, economic investment, and socio‐cultural conditions influence the likelihood that land‐based mitigation potentials are realized. A substantial portion of potential (80%) is in developing countries and LDCs, where feasibility barriers are of greatest concern. Assisting countries to overcome barriers may result in significant quantities of near‐term, low‐cost mitigation while locally achieving important climate adaptation and development benefits. Opportunities among countries vary widely depending on types of land‐based measures available, their potential co‐benefits and risks, and their feasibility. Enhanced investments and country‐specific plans that accommodate this complexity are urgently needed to realize the large global potential from improved land stewardship.

## Graphical abstract

We refine and update the mitigation potentials for 20 land‐based measures in >200 countries and five regions, comparing “bottom‐up” sectoral estimates with integrated assessment models (IAMs). The likely range of cost‐effective (available up to $100/tCO2eq) land‐based mitigation potential is 8–13.8 GtCO2eq yr^−1^ between 2020 and 2050. Mitigation potential varies sixfold across the five regions assessed (0.75–4.8 GtCO2eq yr^−1^) and the top 15 countries account for about 60% of the global potential. Opportunities among countries vary widely depending on types of land‐based measures available, their potential co‐benefits and risks, and their feasibility.

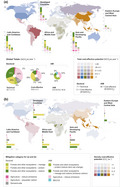

## 1 INTRODUCTION

Land‐based climate mitigation measures, also known as Agriculture, Forestry and other Land Uses (AFOLU) mitigation or natural climate solutions (Griscom et al., 2017)—which if implemented with benefits to human well‐being and biodiversity may also constitute nature‐based solutions—have gained significant attention and importance in public and private sector climate strategies and policies (Seddon et al., 2020). Land‐based measures reduce greenhouse gas (GHG) emissions and/or enhance carbon removals. They include supply‐side interventions in forests and other ecosystems (to protect, manage, and restore), agriculture (to reduce emissions and enhance carbon sequestration), and bioenergy (to reduce fossil fuel emissions and sequester carbon), as well as demand‐side interventions on food waste, diets, and resource use. As of March 2019, 186 countries had included AFOLU measures in their Nationally Determined Contributions (NDCs) under the Paris Agreement, either by specifically listing actions or by including the land sector in their broader GHG reduction targets (Crumpler et al., 2019). Collectively, AFOLU‐related NDC actions make up about 25% of planned GHG reductions in 2030 (Grassi et al., 2017), with most focus on reducing deforestation. Land‐based mitigation measures are also embedded in other international agreements and initiatives, including the Sustainable Development Goals (SDGs), Land Degradation Neutrality (LDN), Aichi Biodiversity Targets, the goals of the New York Declaration on Forests (NYDF), the Bonn Challenge, and the UN Decade on Ecosystem Restoration.

Recent studies estimate that land‐based measures have the potential to mitigate approximately 10–15 GtCO_2_eq yr^−1^ by 2050, corresponding to about 20%–30% of the mitigation needed to achieve the 1.5°C temperature target (Griscom et al., 2017; Jia et al., 2019; Roe et al., 2019; UNEP, 2017). Not only can land‐based measures help close the mitigation gap, if actions are well designed and implemented, mitigation can be delivered in a way that is also cost‐effective, enhances resilience and adaptation to climate change, food security, biodiversity and other ecosystem services, and contributes to international sustainable development goals (Frischmann et al., 2020; Roe et al., 2019; Smith et al., 2019a). Poorly planned and implemented AFOLU mitigation activities, however, entail potential risks and tradeoffs, particularly concerning food security, biodiversity, and water quality and quantity (Humpenöder et al., 2018; Smith et al., 2020).

Achieving climate targets and addressing other land‐related challenges synergistically at national levels remains a large challenge and global progress is lacking. GHG emissions from AFOLU have been increasing since 2000 (Jia et al., 2019). Between 2009 and 2019, policies have only delivered mitigation of about 8 GtCO_2_ from AFOLU, or ~0.5% of total emissions during that period (authors' calculations available in Supplementary Information). Current commitments under the Paris Agreement are more in line with 2.6–3.1°C of warming by the end of the century than the 1.5°C and 2°C committed to in the Paris Agreement (Rogelj et al., 2016). Although some progress has been made, the Aichi Biodiversity Targets for 2020 and the goals of the NYDF, which aimed to halve deforestation and restore 150 million hectares (Mha) by 2020, have not been met, with reversals occurring in some instances since the targets were set (NYDF Assessment Partners, 2020; Secretariat of the Convention on Biological Diversity, 2020). Substantially more resources and effort will therefore be needed to scale‐up land‐based mitigation to fulfill its potential, maximize benefits, and limit tradeoffs.

The efficacy and extent of benefits or risks of land‐based measures largely depend on the type of activity undertaken, deployment strategy (e.g., scale, method, complementarity with other measures and sectors), and geographic context (e.g., current biome dynamics, climate, food system, land ownership) (Smith et al., 2019a). As such, successful and sustainable adoption and appropriate prioritization of land‐based mitigation measures would benefit from more regional and country‐level information on drivers of emissions, mitigation potentials, co‐benefits, and risks (Crumpler et al., 2019). Additionally, realizing AFOLU mitigation and co‐benefit potential will require policies and measures for land and food system management that are location‐ and context‐specific, and adaptable over time (Hurlbert et al., 2019). The success of different policies and implementation of land‐based measures is dependent on enabling conditions and barriers that vary greatly by country. Available funding and economic incentives, governance and institutional capacity, technological capacity, geophysical capacity, socio‐cultural context, and environmental‐ecological conditions all make implementation more or less likely (de Coninck et al., 2018). Accordingly, Parties to the United Nations Framework Convention on Climate Change (UNFCCC) have requested that the Intergovernmental Panel on Climate Change (IPCC) Sixth Assessment Report (AR6) provide more focused assessments of regional mitigation potential and their feasibility. Such information could allow national and international actors to better target investment and effort to areas of promise and need.

This study aims to address the outlined data needs by providing (1) new and/or updated, country‐level technical and cost‐effective (available up to $100/tCO_2_eq) mitigation potentials, using a sectoral approach for 20 land‐based measures; (2) new, regional land‐based mitigation potential estimates generated from the most recent database on integrated assessment models (IAMs); (3) a national feasibility assessment and index as a proxy for gauging the barriers and enabling conditions of implementing land‐based mitigation measures by country; and (4) an analysis of countries by drivers of emissions, mitigation potentials, and feasibility. We compare the available mitigation potentials in the sectoral and IAM approaches, and their feasibility, globally, and across the five high‐level IPCC regions: Africa and Middle East, Asia and Developing Pacific, Developed Countries, Eastern Europe and West‐Central Asia, and Latin America and Caribbean. Based on the mitigation potential and feasibility data, combined with information on emissions drivers, we then provide a framing of countries to highlight different contexts, challenges, opportunities, and priorities for land‐based mitigation.

## 2 METHODS

### 2.1 Mitigation potential

**FIGURE 1:**
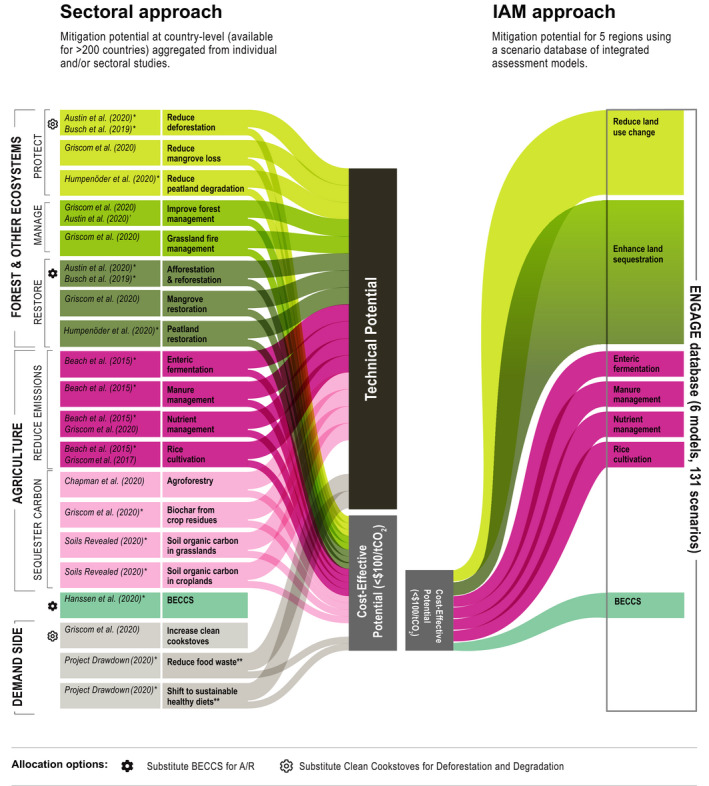
Method and comparison of mitigation potentials using two approaches: Sectoral and IAM. The sectoral approach aggregates 10 studies and 25 datasets, each with a technical and cost‐effective (possible up to $100/tCO_2_eq) potential estimate for 1 of 20 land‐based mitigation measures in >200 countries. The mitigation potentials are averaged over the next 30 years (2020–2050). Data with * represent those that were adapted from their original source, and thus represent new country‐level data. Mean and min‐max range values were used for the five mitigation measures with more than one data source. BECCS and Clean cookstoves are excluded from the aggregate potential to avoid double counting. Demand‐side measures with ** exclude emissions from land‐use change to avoid double counting. Substitution options to calculate total potentials are indicated by symbols. The descriptions and methodologies for each measure are detailed in Table 1. The IAM approach estimates economic mitigation potential in 2050, up to $100/tCO_2_eq in our assessment to compare to the sectoral data. The intermodel median and min‐max range is reported for seven land sector measures from six IAM models and 131 scenario runs in the ENGAGE (Riahi et al., 2021) database. Each IAM measure is described in Section 2.1.2. The flow sizes are illustrative and do not reflect relative mitigation potential sizes; however, the size of the aggregated technical and cost‐effective boxes represent the data. [Correction added on 19 October 2021, after first online publication: Figure 1 has been modified.]

We develop updated global and regional estimates of land‐based mitigation potentials using both sectoral and integrated assessment model (IAM) approaches and compare the results of the two (Figure 1) to establish a likely range of potential. The sectoral approach (also referred to as a “bottom‐up” approach) is based on an extensive literature review and combines mitigation potentials from individual or sectoral studies with available country‐level data, and estimates “technical” potential (possible with available technology, regardless of the cost) and “cost‐effective” economic potential (possible up to $100/tCO_2_eq) in 2020–2050 for 20 land‐based measures in the 250 countries in the IPCC AR6 Working Group III (WGIII) country and region list. We consider mitigation up to $100/tCO_2_eq as cost‐effective as it is in the middle of the range for carbon prices in 2030 for a 1.5°C pathway, and at the low end of the range in 2050 (Rogelj et al., 2018)—the timeline that we target in this assessment. Since technical potentials may not be plausible or desirable due to economic, social, political, or environmental constraints and tradeoffs, we focus the regional assessment on cost‐effective potentials which represent a more realistic level regarding public willingness to pay for climate mitigation. We do not provide sectoral estimates for other carbon prices as there were fewer available data. The IAM approach (sometimes referred to as a “top‐down” approach) adapts land sector data from the most recent IAM intercomparison database, ENGAGE (Riahi et al., 2021), and estimates cost‐effective potential (possible up to $100/tCO_2_eq) in 2050 across six models and 131 scenarios for seven land‐based measures in five regions. IAMs estimate economic potentials using carbon prices; therefore, we do not provide technical potential estimates from IAMs.

IAMs assess the mitigation potential of multiple and interlinked practices across sectors and regions and can therefore account for interactions and tradeoffs (including land competition, use of other resources, and international trade) between them. IAMs can also optimize across mitigation measures based on market effects and costs. A few sectoral models also consider some level of inter‐ and cross‐sector interactions and land allocation; however, when aggregating potentials across sectoral estimates with different methods, it is difficult to completely account for land competition and avoid double counting. Since land‐based mitigation is relatively new in IAMs (Popp et al., 2017), only a limited portfolio of land‐based mitigation measures is included (Figure 1). IAM data also generally have coarser resolution compared to sectoral estimates, and as such, sectoral estimates may provide more robust mitigation estimates, including country‐level estimates for individual measures. To provide a comprehensive understanding of land‐based mitigation potentials and their likely ranges and boundaries, it is therefore helpful to assess and compare both types of approaches and estimates (Figure 1). We use the sectoral estimates as the primary method in the regional mitigation assessment and feasibility (Section 3.2) given the country‐level disaggregation and availability of more mitigation measures. The two approaches are described in more detail in Sections 2.1.1 and 2.1.2.

#### 2.1.1 Sectoral estimates

To assess national and regional mitigation potentials across a wide suite of land‐based measures, we compiled and developed both technical and cost‐effective (possible up to $100/tCO_2_eq) mitigation potentials implemented between 2020 and 2050 (averaged) using the best available data with country‐level resolution. The mitigation potential quantified in the 20 measures include reductions and removals of CO_2_ and reductions of N_2_O and CH_4_. The mitigation potentials are derived from individual and/or sectoral studies and datasets which use a range of methods, including sectoral economic modeling, optimization modeling, and spatial analysis (the definitions and methods for each of the 20 mitigation measures are outlined in Table 1). Indirect impacts such as the substitution effects of bioenergy, biochar, and wood products on fossil fuel emissions are excluded due to a lack of country‐level data. However, we provide a global potential estimate for displacing fossil fuels with BECCS, biochar, and biogas from manure management (highlighted in Figure 3). For BECCS, we estimated cost‐effective potential for avoided fossil fuel emissions by comparing the carbon contents and bioenergy use of projected energy systems across a standardized set of baseline and carbon price scenarios at 100$/tCO_2_ (Bauer et al., 2020), and technical potential using the baseline carbon contents of the electricity system. For biochar and biogas from manure management (anaerobic digesters), avoided fossil fuel emissions were estimated using the same energy system emissions intensities as for BECCS, assuming electricity is the primary product.

**TABLE 1:** Definitions and methods for estimating the technical and cost‐effective mitigation potentials for 20 land‐based mitigation measures using a sectoral approach

Mitigation category	Mitigation measure	Definition	Method
Forests and other ecosystems—protect	Reduce deforestation	Avoided emissions from deforestation (forests are defined as 30% or greater tree cover)	Adapted data from Busch et al. (2019), which used a spatially explicit pantropical marginal abatement cost curve model and estimated the mitigation potential of avoided emissions from deforestation between 2020 and 2050 in the tropics. Technical potential was calculated as avoiding all business‐as‐usual (BAU) deforestation and cost‐effective potential was calculated as the difference between BAU and $100/tCO_2_eq. Carbon price values are in constant USD 2014. Area estimates were extracted from the spatial maps in the model.
		Avoided emissions from deforestation (forests as defined in (FAO, 2020b)	Adapted data from Austin et al. (2020), which used the Global Timber Model (GTM), a dynamic economic forest model to estimate global forest sector mitigation potential (mean of avoided annual emissions between 2015 and 2050). Technical potential was calculated as avoiding all BAU deforestation, and cost‐effective potential was calculated at a carbon price below $100/tCO_2_eq compared to baseline levels. Carbon price values are in constant USD 2017. Forest area estimates for tropical countries were obtained from FAO, 2020 and country‐level inventories in temperate countries from Tian et al. (2018)
	Reduce mangrove loss	Avoided emissions from degradation and/or anthropogenic loss of carbon stocks in mangrove ecosystems	Data from Griscom et al. (2020), expanded to include non‐tropical countries, calculates the extent of baseline degradation and/or conversion based on an estimate of current mangrove extent, and recently reported loss rate from 1996 to 2016. Mangrove carbon stocks are calculated by combining the mean of seven aboveground and belowground vegetation biomass estimates from the literature with the most recent and comprehensive global estimate of SOC density in the top meter. Cost‐effective potential (<$100/tCO_2_eq) was estimated as 90% adoption of technical potential, following Griscom et al. (2017). The area data were extracted from Global Mangrove Watch (GMW, 2019) for 1996, 2007, 2010, and 2016 timesteps to calculate rates of mangrove loss (Bunting et al., 2018).
	Reduce peatland degradation	Avoided GHG emissions (CO_2_, CH_4_, and N_2_O) from degradation of intact peatlands (does not include conversion of vegetation)	Adapted data from (Humpenöder et al., 2020), which used MAgPIE, a spatially explicit economic land model, to develop new country‐level mitigation potential estimates of peatland protection (avoided degradation) in the context of a 2℃ transformation pathway. The economic mitigation potential of peatland protection was calculated as the difference between SSP2_RCP2p6+PeatProt (land‐based climate policy incl. peatland protection)—SSP2_Ref (no climate policy) in the year 2035 (<$100/tCO_2_eq). The estimate of the technical potential for peatland protection is based on an SSP3_Ref scenario (no climate policy), in which degraded peatland increases from 45.8 Mha in 2015 to 56.4 Mha in 2050 at the global level. We assume that the associated GHG emissions from this additional loss of intact peatland (10.6 Mha) could be avoided by stringent peatland protection from 2015 onwards. GWP100 values from AR5 (CH_4_=28, N_2_O=265) were used to convert non‐CO_2_ gases into CO_2_eq.
Forests and other ecosystems—manage	Improve forest management	Avoided emissions and enhanced sequestration from improved natural forest management, including reduced‐impact logging, extended harvest rotations, increased post‐harvest sequestration rates, and designation of set‐aside areas for protection from logging activity	Data from Griscom et al. (2020) for tropical countries and Griscom et al. (2017) for non‐tropical countries. Griscom et al. (2020) estimated country‐level biophysical (technical) potential based on avoidable selective logging emissions in natural forests reported by Ellis et al. (2019). Ellis et al. (2019) estimate country‐level baseline pantropical selective logging emissions and calculate the portion of these emissions that could be avoided through implementing reduced‐impact logging for climate practices (RIL‐C). Griscom et al. (2017) estimated the additional carbon sequestration potential of native forests under non‐intensive management for wood production. Cost‐effective potential (<$100/tCO_2_eq) was estimated as 60% of technical potential, following Griscom et al. (2017).
		Enhanced carbon sequestration from improved forest management activities	Adapted data from Austin et al. (2020), which used the Global Timber Model (GTM), a dynamic economic forest model to estimate global forest sector mitigation potential (mean of avoided emissions between 2015 and 2050). Technical potential was calculated at a constant carbon price of $2,000/tCO_2_eq to stimulate the maximum available carbon in the model. Cost‐effective potential was the carbon sequestration potential given for scenarios with a carbon price below $100/tCO_2_eq in 2050 compared to baseline levels. Carbon price values are in constant USD 2017. Forest area estimates for tropical countries were obtained from FAO (2020b) and country‐level inventories in temperate countries from (Tian et al., 2018).
	Grassland fire mgmt	Avoided emissions from grasslands fires	Data from Griscom et al. (2020), expanded to include non‐tropical countries, takes country‐level biophysical (technical) potential from Lipsett‐Moore et al. (2018), and applies a uniform global cost constraint from Griscom et al. (2017). Estimates primarily reflect N2O and CH4 emissions, since most CO_2_ emissions from grassland fires are re‐sequestered by re‐growth within a few years. Cost‐effective potential (<$100/tCO_2_eq) was estimated as 30% adoption of technical potential, following Griscom et al. (2017). Area estimates were determined as savanna habitat within the Global Fire Emissions Database pixels with >600mm annual precipitation and positive emissions abatement potential, as defined by Lipsett‐Moore et al. (2018).
Forests and other ecosystems—restore	Afforestation and Reforestation	Carbon sequestration by shifting from non‐forest cover to forest cover at 30% tree cover threshold with a region‐specific mix of plantation forestry and natural forest regrowth	Adapted data from Busch et al. (2019), which used a spatially explicit pantropical marginal abatement cost curve model and estimated the mitigation potential of mean annual additional sequestration over the time period 2020–2050. Technical potential was calculated as the enhanced removals at <$500/tCO_2_eq relative to the business‐as‐usual (BAU) scenario, and cost‐effective potential was calculated as at $100/tCO_2_eq relative to BAU. Carbon price values are in constant USD 2014. Area estimates were extracted from the spatial maps in the model.
		Carbon sequestration from afforestation and reforestation (forests as defined in FAO (2020b))	Adapted data from Austin et al. (2020), which used the Global Timber Model (GTM), a dynamic economic forest model to estimate global forest sector mitigation potential (mean of avoided emissions between 2015 and 2050). Technical potential was calculated at a constant carbon price of $2,000/tCO_2_eq to stimulate the maximum available carbon in the model. Cost‐effective potential was the carbon sequestration potential given for scenarios with a carbon price below $100/tCO_2_eq in 2050 compared to baseline levels. Carbon price values are in constant USD 2017. Forest area estimates for tropical countries were obtained from FAO (2020) and country‐level inventories in temperate countries from Tian et al. (2018)
	Mangrove restoration	Carbon sequestration from restoring mangroves lost since 1996, after excluding those converted to urban land or lost to erosion.	Data from Griscom et al. (2020), expanded to include non‐tropical countries, calculates the potential restorable mangrove area from the area of mangrove cover lost since 1996, after subtracting the area converted to urban land, or eroded, as these two categories of loss were assumed to not be feasible for restoration. Carbon sequestration potential from restoration was calculated by multiplying the country‐level area of restorable mangroves by a global carbon sequestration value of 6.4 and converting to CO_2_ equivalent using a conversion factor of 3.67. The proportion of cost‐effective (<$100/tCO_2_eq) potential (30% adoption of technical potential) was estimated following Griscom et al. (2017), who derived marginal abatement cost (MAC) curves from a large project database in Bayraktarov et al. (2016). The area data were extracted from the spatial maps used to calculate potentials.
	Peatland restoration	Avoided GHG emissions (CO_2_, CH_4_, and N_2_O) from restoration (re‐wetting) of degraded peatlands	Adapted data from (Humpenöder et al., 2020), which used MAgPIE, a spatially explicit economic land‐use model, to develop new country‐level mitigation potential estimates of peatland protection and restoration in the context of a 2℃ transformation pathway. The economic mitigation potential of peatland restoration was calculated as the difference between SSP2_RCP2p6+PeatRestor (land‐based climate policy including peatland protection +restoration) and SSP2_RCP2p6+PeatProt (land‐based climate policy including peatland protection) in the year 2035 (<$100/tCO_2_eq). The estimate of the technical potential for peatland restoration is based on the assumption that all presently degraded peatlands (45.8 Mha in 2015 globally) would be rewetted. GWP100 values from AR5 (CH_4_=28, N_2_O=265) were used to convert non‐CO_2_ gases into CO_2_eq.
Agriculture—reduce emissions	Enteric fermentation	Avoided CH_4_ emissions from ruminant livestock enteric fermentation through improved feed conversion, antibiotics, bovine somatotropin (bST), propionate precursors, antimethanogens, and intensive grazing	Adapted data from Beach et al. (2015b) as extended through 2050 in USEPA (2019), which estimate technical potential as net changes in CH4 emissions from the global adoption of six mitigation options (listed in definition). Global livestock populations were allocated across livestock production systems in each country based on data from the Food and Agriculture Organization (FAO) and mitigation options were applied where it was technically feasible (applied only to the portion of each livestock type assessed—beef cattle, dairy cattle, goats, and sheep—that are intensively managed). Cost‐effective mitigation potential was calculated based on the quantity of mitigation available from options with break‐even prices at or below $100/tCO_2_eq (using USD 2017 constant carbon price values). GWP100 values from AR4 (CH_4_=25) were used to convert CH_4_ into CO_2_eq.
	Manure management	Avoided CH_4_ and N_2_O emissions from livestock manure management in anaerobic systems through incorporation of small‐scale or large‐scale anaerobic digesters.	Adapted data from Beach et al. (2015b) as extended through 2050 in USEPA (2019), which estimate technical potential from net changes in CH4 and N2O emissions associated with global adoption of different types of anaerobic digesters to manage manure from pigs and dairy cattle. Large‐scale anaerobic digester systems were assumed to be technically feasible only in intensively managed dairy cattle and pig production systems. Small‐scale digesters suitable for managing waste from several animals were assumed to be available only in extensively managed dairy cattle and pig production systems in lower‐income countries. Cost‐effective mitigation potential was calculated based on the quantity of mitigation available from options with break‐even prices at or below $100/tCO_2_eq (using USD 2017 constant carbon price values). GWP100 values from AR4 (CH_4_=25, N_2_O=298) were used to convert non‐CO_2_ gases into CO_2_eq.
	Nutrient management	Avoided N_2_O and CH_4_ and changes in carbon sequestration in cropland soils associated with nitrogen application through changes in fertilizer application and management practices: split fertilization, 100% crop residue incorporation, nitrification inhibitors, and reducing nitrogen fertilizer applications by 20%.	Adapted data from Beach et al. (2015b) as extended through 2050 in USEPA (2019), which estimates technical potential from net changes in GHG emissions associated with global adoption of mitigation options (listed in definition) to reduce emissions from nutrient management on croplands. The DAYCENT crop process model was used to calculate biophysical values including changes in crop yields, N_2_O and CH_4_ emissions, and soil organic carbon (SOC) sequestration for barley, maize, sorghum, soybeans, and wheat on a 25 km^2^ global grid basis. Cost‐effective mitigation potential was calculated based on the quantity of mitigation available from options with break‐even prices at or below $100/tCO_2_eq (using USD 2017 constant carbon price values). GWP100 values from AR4 (CH_4_=25, N_2_O=298) were used to convert non‐CO_2_ gases into CO_2_eq. It was assumed that cropland areas can adopt only one mitigation measure, though in practice it may be feasible to adopt multiple options simultaneously. The set of technically feasible options that resulted in a net reduction in GHG emissions for a given crop/country combination were each applied to an equal share of land area for that crop/country. Thus, these estimates of mitigation potential may be conservative.
		Avoided N_2_O emissions (direct and indirect) and production‐linked CO_2_ emissions from reducing total fertilizer application through the use of best practices and/or improved technologies	Data from Griscom et al. (2020), expanded to include non‐tropical countries, estimate country‐level mitigation potential for improved cropland nutrient management practices based on optimizing the efficiency of nitrogen inputs relative to nitrogen harvested in products. Savings in nitrogen fertilizer consumption per country were estimated from projections of BAU and optimized consumption in 2030, assuming fertilizer efficiency can be raised to regional targets identified in Zhang et al. (2015). Estimates include direct and indirect reductions in N_2_O emissions as well as upstream CO_2_ emissions from fertilizer manufacture. GWP100 values from AR4 (N_2_O=298) were used to convert N_2_O into CO_2_‐eq. Cost‐effective potential (<$100/tCO_2_eq) was estimated as 90% of technical potential, based on Griscom et al. (2017). The applicable area in each country is assumed to be the total cropland area in 2018 as reported by FAO, (2020a).
	Rice cultivation	Avoided CH_4_ and N_2_O emissions and enhanced soil organic carbon sequestration from rice cultivation through: nutrient management (reduced or optimized nitrogen fertilizer application, use of slow release fertilizer, application of nitrification inhibitors, switching from urea to ammonium sulfate), residue management (100% incorporation), water management (midseason drainage, alternate wetting and drying, switching from irrigated to dryland rice), dry seeding, tillage strategies, and combinations of these activities	Adapted data from Beach et al. (2015b) as extended through 2050 in USEPA (2019), which estimate technical potential from net changes in GHG emissions associated with the global adoption of mitigation options to reduce emissions from rice cultivation (listed in definition). The DeNitrification‐DeComposition (DNDC) crop process model was utilized to estimate changes in rice yields, CH_4_ and N_2_O emissions, and soil organic carbon sequestration. The DNDC model includes data on global rice production that enabled characterization of the baseline distribution of water management and seeding practices. Thus, mitigation was estimated relative to baseline practices in each rice‐producing country. Cost‐effective mitigation potential was calculated based on the quantity of mitigation available from options with break‐even prices at or below $100/tCO_2_eq (using USD 2017 constant carbon price values). GWP100 values from AR4 (CH_4_=25, N_2_O=298) were used to convert non‐CO_2_ gases into CO_2_eq.
		Avoided CH_4_ and N_2_O emissions associated with anaerobic decomposition by employing the periodic draining of rice soils and the removal of rice residues in flooded and upland rice production lands	Data from Griscom et al. (2017), which assumed an average 35% reduction in combined CO_2_‐equivalent emissions from using improved rice management practices. GWP100 values from AR4 (CH_4_=25, N_2_O=298) were used to convert non‐CO_2_ gases into CO_2_‐eq. Country‐level estimates were then derived by applying the reduction to rice‐derived CO_2_‐equivalent emissions per country in 2030 as projected by USEPA (2013). Cost‐effective mitigation (<$100/tCO_2_eq) was estimated as 60% adoption of technical potential, following Griscom et al. (2017) which is based on averages of Golub et al. (2009), Beach et al. (2015a), and USEPA (2013).
Agriculture—sequester carbon	Agroforestry	Carbon sequestration from adding aboveground woody carbon storage in agriculture systems (crop and pasture pixels with <25% tree cover)	Adapted data from Chapman et al. (2020), which estimate the potential aboveground carbon contributions of trees integrated into agricultural lands (defined as crop and pasture pixels with <25% tree cover to avoid potential overlap with A/R) to develop new country‐level mitigation potential estimates for agroforestry. The median aboveground carbon density in crop and pasture land pixels with >5 MgC ha^−1^ in each country‐biome combination was calculated as a benchmark and that value was applied across a range of area percentages of the agricultural area in that country‐biome region with little to no standing trees (<5 MgC ha^−1^). Soil organic carbon changes were not considered. We estimated the technical sequestration potential using a 50% adoption scenario and cost‐effective sequestration potential using a 10% adoption scenario which is a proxy for mitigation at $100/tCO_2_eq (based on Griscom et al., 2017). Sequestration rates were calculated assuming a 30‐year horizon to meet the area‐based 50% and 10% potential scenario of CO_2_ sequestration. Area values were extracted from the spatial map outputs. For technical sequestration potential, the total area of cropland was considered. For cost‐effective sequestration potential, 20% of the crop area was considered.
	Biochar from crop residues	Enhanced carbon sequestration by amending agricultural soils with biochar, which increases the agricultural soil carbon pool by converting rapid‐mineralizing carbon (crop residue biomass) to persistent carbon (charcoal) through pyrolysis.	Developed new estimates, expanding analysis from Griscom et al. (2017) which estimated mitigation potential of biochar production considering crop residues only as a subset of possible feedstocks. We expanded the analysis to include primary and secondary residues of the top 15 crops globally by area harvested, plus cocoa, coconut, and potatoes as important regional crops (FAOSTAT, 2021), using residue production ratios from the literature (Bentsen et al., 2014; Fischer et al., 2007; Koopmans & Koppejan, 2007; Scarlat et al., 2010; Searle & Malins, 2015; Syamsiro et al., 2012). The fraction of primary residues of each crop that could be sustainably removed from fields was estimated based on Scarlat et al. (2010) and Woolf et al. (2010), and the fraction required for animal feed was then deducted from available residues at a regional level based on Herrero et al. (2013). Residue carbon content and biochar yield were based on Griscom et al. (2017). It was assumed that 80% of biochar carbon persists on a timescale of >100 years (IPCC, 2019). We also include avoided N_2_O emission from pyrolysis of residues that would otherwise decay in the field, assuming nitrogen content per crop based on IPCC (2019) and the Phyllis database (Phyllis^2^, ^2021^), and a 1% emission factor (IPCC, 2019). For rice residues, we also consider avoided methane emission from decomposition of straw in paddies based on IPCC 2019, assuming an additional 37% reduction in methane beyond that achieved by rice management practices (Proville et al., 2020). The cost‐effective potential (<$100/tCO_2_eq) by country was estimated using the cost–benefit model and assumptions of (Woolf et al., 2016) and global residue supply curves given by (Daioglou et al., 2016), including revenue from sale of energy and from carbon credits but excluding impacts on crop yield. Applicable area and density calculated by discounting total harvested area of target crops (FAOSTAT, 2021) by the fraction of total cropland in each country under multiple cropping extracted from Waha et al. (2020).
	Soil organic carbon in croplands	Enhanced soil organic carbon sequestration by shifting from current management to no‐till management with an input scenario consistent with cover cropping.	Adapted data from Soils Revealed (2020) which calculates the annual rate of change in soil organic carbon (SOC) stocks based on Tier 1 stock difference approach (IPCC, 2019), to develop new estimates of technical potential and cost‐effective potential ($100/tCO_2_eq) for croplands and grasslands. For the technical potential, SOC is defined relative to the reference SOC stock (SOCref) for a given location by a combination of linear SOC‐modifying factors for land use (FLU), management (FMG), and input levels (FI): SOCt = SOCref × FLU,t × FMG,t × FI,t. Thus, ΔSOC will be non‐zero only if at least one of land‐use, management or inputs differs between the start and end of the 20‐year accounting period, at which time most of the SOC accrual has occurred. Current land use (FLU) was defined by reclassifying the ESA’s Climate Change Initiative Land Classification (CCI LC) map for the year 2018 using an overlay of rice production and ley forage production from Monfreda et al. (2008), forest, wetland, urban/built up, grassland, cropland (non‐paddy rice), paddy rice, and managed pasture. Wetland and urban areas are not considered in this analysis. Current cropland FMG was assumed to represent conventional tillage everywhere. Current grassland FMG was defined based on soil degradation level from The Global Assessment of Soil Degradation (GLASOD) map (Oldeman et al., 1991). For croplands, FI was defined as low where >50% of crop residues are used as animal forage based on Wirsenius (2003) or where >50% of area harvested is allocated to vegetable or fiber crops according to Monfreda et al. (2008). Remaining cropland FI was set to medium. These conditions then defined the 2018 SOC stock levels at each pixel. Future SOC stocks for the year 2038 were then calculated at each pixel using the SOC‐modifying factors associated with the assumptions listed in each scenario. Then, for each pixel, ΔSOC was calculated as the difference between 2038 and 2018 stocks divided by 20 years. This unconstrained technical potential was then reduced using a climatic constraint on cropping interventions—for tillage, tropical montane, tropical wet and polar climates were masked out; for cover crops, tropical dry, warm/cool temperate dry, boreal and polar climates were masked out. Area available was further constrained by current regional adoption rates of no‐till‐based conservation agriculture adoption (Prestele et al., 2018) and winter crops (Siebert et al., 2010). In this constrained cropping area, we assumed 90% adoption for the cost‐effective potential at $100/tCO_2_eq (based on Griscom et al. (2017)). For grasslands, the technical potential was constrained to degraded grasslands and non‐degraded grasslands (defined by GLASOD) were masked out due to lack of opportunity. We assumed 60% adoption to estimate cost‐effective potential (<$100/tCO_2_eq) based on Griscom et al. (2017).
	Soil organic carbon in grasslands	Enhanced soil organic carbon sequestration in managed pastures, by shifting from current practices to improved sustainable management with light to moderate grazing pressure and at least one improvement. For rangelands, a shift from current management defined by land degradation to nominally managed.	
Bioenergy	BECCS	Carbon sequestration from electricity generation derived by combusting lignocellulosic crop‐based biomass (Miscanthus, switchgrass, short‐rotation coppiced trees such as poplar and Eucalyptus) and combined with carbon capture and storage. This excludes the energy substitution effect	Adapted data from Hanssen et al. (2020), which estimates technical potential from the amount of net negative CO_2_ emissions that can biophysically be achieved over a 30‐year evaluation period, while considering all relevant flows of GHGs, including land‐use change emissions, the lost carbon sequestration capacity of natural vegetation (“foregone sequestration”), bioenergy supply chain emissions including fertilizers, CO_2_ capture efficiency, and CO_2_ that is sequestered through carbon capture and storage (CCS). Alternative uses of biomass crops such as for pyrolysis or direct biomass burial were not considered. Furthermore, we exclude the mitigation potential from the substitution of fossil fuels that occurs from producing the bioenergy. We develop new estimates of cost‐effective mitigation potential up to $100/tCO_2_eq by adding costs for biomass production, and conversion to electricity combined with CCS (Daioglou et al., 2019; Krey et al., 2019). These costs are based on the IMAGE integrated assessment model on an SSP2 baseline, with biomass costs calculated on a 0.5° × 0.5° grid, and conversion and capture costs for 26 global regions. Carbon stock changes and biomass yields are based on the LPJml global vegetation model and are supplemented with literature‐derived yield calibration factors and supply chain emissions. Land availability assumed in determining biophysical potential is constrained by excluding projected urban and agricultural land (cropland and pastures according to an SSP2 land‐use projection of the IMAGE model as presented in Doelman et al. (2018)), as well as areas with low bioenergy crop yields (<5% of global maximum) or no potential to deliver net negative emissions through BECCS. Our BECCS estimates only represent the potential from energy crop plantations as we did not consider the potential of BECCS which can be achieved using agricultural and forestry residues.
Demand‐side	Increase clean cookstoves	Avoided emissions due to the introduction of improved cookstoves which leads to reduced harvest of wood fuel used for cooking and heating	Data from Griscom et al. (2020) which developed country‐level technical potentials based on Bailis et al. (2015), applying a 49% potential reduction to the national emissions from unsustainable wood fuel estimated by the latter, and excluding potential wood fuel that could have arisen as a by‐product of other land‐use change. Cost‐effective mitigation (<$100/tCO_2_eq) was estimated as 30% of technical potential, following Griscom et al. (2017).
	Reduce food waste	Emissions reductions from diverted agricultural production (excluding land‐use change) from reduced food loss and wastage from all stages of production, distribution, retail, and consumption through the implementation of measures such as improved storage and transport systems, generation of public awareness, and changing consumer behaviors.	Adapted data from Project Drawdown (2020) and developed new country‐level estimates for technical potential and cost‐effective potential of reducing food waste. Total food loss and wastage is calculated according to regional estimates of waste generated at each supply chain stage projected to 2050 (FAO, 2011, 2019), applied to aggregated country‐level food demand by commodity type. The technical potential was estimated as emissions reductions from the incremental reduction of food waste until 75% reduction is achieved in 2050, applied across all stages of the supply chain. The cost‐effective potential assumes a 50% reduction in food waste by 2050. The resulting reductions represent the total global reduction from avoided agricultural production and does not include emissions reductions from avoided land conversion and ecosystem protection to avoid double counting. GWP100 values from AR4 (CH_4_=25, N_2_O=298) were used to convert into CO_2_eq.
	Shift to sustainable healthy diets	Emissions reductions from diverted agricultural production (excluding land‐use change) from the adoption of sustainable healthy diets: (a) maintain a 2250 calorie per day nutritional regime; (b) converge to healthy daily protein requirement, limiting meat‐based protein consumption to 57 grams/ day; and (c) purchase locally produced food when available.	Adapted data from Project Drawdown (2020) and developed new country‐level estimates for technical potential and cost‐effective potential of shifting to healthy diets. Technical potential is estimated as the difference between the emissions from projected baseline country‐level dietary trends and emissions with a 75% global adoption of a sustainable and healthy diet (components listed in definition), averaged over the years 2020–2050. The cost‐effective potential assumes a 50% adoption of a healthy diet. Adoption scenarios in this model grow linearly over time starting from the base year of 2014 and are considered “complete” in 2050. The resulting reductions represent the total global reduction from avoided agricultural production and does not include emissions reductions from avoided land conversion and ecosystem protection to avoid double counting. GWP100 values from AR4 (CH_4_=25, N_2_O=298) were used to convert into CO_2_eq.

Estimates represent individual studies or sectoral models (referred to as “bottom‐up” or sectoral approaches). Cost‐effective mitigation represents economic mitigation possible up to $100/tCO_2_eq. The definitions of each measure include whether the estimate accounts for emission reductions, carbon sequestration, or both.

Our work builds on and advances previous global studies (Fuss et al., 2018; Griscom et al., 2017; Jia et al., 2019; Roe et al., 2019; Smith et al., 2013, 2014; UNEP, 2017) and regional studies (Griscom et al., 2020; Roe et al., 2019) on land‐based mitigation potentials. Specifically: (1) Using existing studies or models, we developed new country‐level mitigation estimates on agroforestry, biochar, peatland degradation, peatland restoration, soil organic carbon enhancement in croplands and grasslands, reduced food waste, and shifts to healthy diets; (2) we adapted existing global mitigation estimates and created country‐level cost‐effective mitigation potentials for: reduced deforestation, afforestation/reforestation (A/R), forest management, enteric fermentation, manure management, crop nutrient management, rice cultivation, and bioenergy with carbon capture and storage (BECCS); (3) we expanded the country‐level data published by Griscom et al. (2020) to provide global coverage where relevant; (4) we developed data on land area (hectares) associated with mitigation potentials; and (5) we calculated “mitigation density” potentials (cumulative technical mitigation between 2020 and 2050 divided by total land area used) for each mitigation measure by country. For measures with more than one dataset, we provided a range and calculated average mitigation potentials for the aggregate estimates.

As much as possible, elements of the analysis were designed to avoid potential double‐counting of mitigation opportunities. When aggregating total sectoral potentials, we excluded measures that may overlap on the same land. To avoid double counting with reduced deforestation, we excluded increased clean cookstoves as they may also reduce emissions from avoided forest loss and degradation. We included demand‐side measures, shifting to healthy diets and reduced food waste in the aggregate estimate; however, we only account for the GHG reductions from diverted agricultural production, and exclude emissions reductions associated with land‐use change. To avoid double counting with A/R and biochar, we also excluded BECCS. We included reduced peatland degradation and peatland restoration as the mitigation potential in our estimates do not account for vegetation impacts (deforestation and reforestation), but rather, avoided emissions from draining and rewetting. We selected reduced deforestation and A/R over the excluded activities given their scale and geographic scope; however, a different allocation could also be chosen (Figure 1).

#### 2.1.2 IAM estimates

We assessed cost‐effective land‐based mitigation potentials from the most recent IAM database, ENGAGE (Riahi et al., 2021), and where relevant, additional scenarios based on recent model versions (see Supplementary Information), for six integrated assessment models with available land sector data (AIM‐Hub (Fujimori et al., 2014), IMAGE (Stehfest & Planbureau voor de Leefomgeving, 2014), MESSAGEix‐GLOBIOM (Huppmann et al., 2019), POLES (Criqui et al., 2015), REMIND‐MAgPIE (Kriegler et al., 2017; Luderer et al., 2013), and WITCH‐GLOBIOM (Bosetti et al., 2006; Emmerling et al., 2016). We calculated the potential as the emission reduction and/or carbon enhancement available at a carbon price of $100/tCO_2_eq (range between $50 and 150/tCO_2_eq) compared to the “No Policy” baseline scenario (“NPi2100” baseline for AIM and POLES models) in 2050. As IAM mitigation estimates are based on a set of policy scenarios that result in a range of carbon prices over time, we included the range between $50 and $150/tCO_2_eq in 2050 to best represent mitigation at $100/tCO_2_eq (approximate median) across IAMs. Although IAM and sectoral approaches do not use the exact same carbon pricing method, they are close approximations of cost‐effective potential. We chose the 2050 time horizon to be more comparable to sectoral estimates as model assumptions delay a majority of land‐based mitigation to mid‐century. We report the intermodel weighted median and range mitigation potential values across 131 scenarios and 6 models at a global and regional (five regions) level. We use a weighted median to avoid biasing estimates towards models with more scenario runs. The weighted median compared to the natural median produced slightly lower non‐CO_2_ mitigation and slightly higher BECCS mitigation. GWP100 values from AR5 (CH_4_ = 28, N_2_O = 265) were used by the IAMs to convert non‐CO_2_ gases into CO_2_eq.

Seven land‐based mitigation measures comparable to our sectoral list were available to extract across the IAMs: (1) reduce land‐use change (“CO_2_ positive | Land Use”); (2) A/R (“Carbon sequestration | Land Use | Afforestation”); (3) enteric fermentation (“CH4 Agriculture | Enteric fermentation”); (4) manure management (“CH4 + N2O Agriculture | Manure mgmt.”); (5) rice cultivation (“CH4 Agriculture | Rice”); (6) crop nutrient management (N2O Agriculture | “Managed soils”); and (7) BECCS (“Carbon sequestration | CCS | Biomass”). We also report carbon sequestration from land use (Figure 1) which includes all land‐based carbon sequestration including A/R (e.g., forest management and regrowth). For A/R, we provide two mitigation estimates, the first using the same “No Policy” baseline as the other IAM mitigation estimates (which already deploys some A/R), and the second using no baseline to illustrate the full A/R potential and more easily compare to the sectoral studies. When aggregating the CDR and total land‐based mitigation values, we use the first A/R mitigation potential estimate to maintain consistency with the other measures. Baselines and their assumptions differ across the models and can have a large effect on mitigation potentials. For example, some baseline scenarios assume low carbon prices and thus already include some emission reductions, which reduces the additional mitigation potentials when comparing a strong mitigation scenario to the respective baseline. Similar to the sectoral estimates, the IAMs considered in this assessment only account for direct GHG emissions reductions or removals and do not include indirect substitution effects on fossil fuel emissions.

### 2.2 Feasibility assessment

The global shift needed to limit warming to 1.5°C or 2°C will require a range of enabling conditions to catalyze action and adequately address the synergies and tradeoffs between mitigation and sustainable development (IPCC, 2018). The enabling conditions, or feasibility, of effectively implementing mitigation measures, are highly contextual and vary according to each country's circumstances. We developed a quantitative index as a proxy for country‐level feasibility to implement actions and realize mitigation potentials. Our framework is based on the IPCC’s definition of feasibility, defined as the capacity of a system to attain a specific outcome (de Coninck et al., 2018), and includes six dimensions of feasibility: economic, institutional, geophysical, technological, socio‐cultural, and environmental‐ecological feasibility. Given the broad scope of “feasibility,” we considered a range of enabling conditions across the six dimensions, including both state capacity and private sector/land‐owner enabling conditions across all land‐use management types. Our feasibility index represents a first attempt to quantify country‐level feasibility using the IPCC’s qualitative feasibility assessment framework. The resulting feasibility index is intended to illustrate where mitigation potential and feasibility are correlated, and identify gaps that can be addressed to increase likelihood of implementation. Where more detailed regional data exist, the approach can be refined. The feasibility assessment consisted of a two‐part literature review followed by expert review of the datasets found, harmonization and scaling, and finally, calculation of a feasibility score for each country.

#### 2.2.1 Literature review

A preliminary literature review identified the most important enabling conditions and barriers for land‐based mitigation actions. A list of feasibility factors was drawn from this literature review, which included a broad range of empirical and theoretical studies across activities in the AFOLU sector. Factors were categorized under one of the six abovementioned IPCC dimensions of feasibility. A second literature review identified quantitative datasets describing the enabling conditions and barriers previously documented as relevant.

#### 2.2.2 Expert review and indicator selection

We evaluated the quality of the datasets to determine the country coverage and to highlight potential correlations among potential feasibility factors. For the final selection of indicators (Table 2), feasibility factor candidates were required to meet a minimum of two specific criteria. First, indicator data should be available from the last 5 years for a sufficient number of countries (>100) to make a meaningful assessment. Second, a clear logic should exist in the direction of the relationship between the variable in question and the feasibility of implementation of a mitigation measure. For instance, increased tenure insecurity is associated with greater difficulty in implementing land‐use activities in the AFOLU sector (Djenontin et al., 2018; Robinson et al., 2014). To incorporate more detailed enabling factors, we included some indicators that apply to the feasibility of implementing mitigation activities in either agriculture or forests and other ecosystems (agricultural value added, agriculture total factor productivity, and forest rents), recognizing that they may not necessarily apply to the other. Variables that exerted either an unclear or mixed effect (e.g., subsidies in the agriculture sector) were excluded. These two criteria resulted in the selection of 19 feasibility indicators (Table 2).

**TABLE 2:** Indicators (19) used in the feasibility assessment

IPCC Feasibility dimensions	Indicators and justification	Sources	Year	Number of countries	References
Economic	**Gross domestic product (GDP) per capita, converted by purchasing power parity (PPP) conversion factor (constant 2017 international $)**. The implementation costs of mitigation measures will be easier to bear for countries with stronger economic capacity.	World Bank ICP, 2020	2018	188	Jewell & Cherp, 2019
	**Forest rents ($/ha of forest area)** calculated as forest rents multiplied by GDP, PPP (constant 2017 international $) divided by forest area (hectares). Countries with a strong forest sector will face fewer barriers implementing AFOLU implementations and will have a stronger strategic interest to invest in the forest sector.	World Bank, 2011, 2020; FAO	2016	182	Bustamante et al., 2019
	**Agricultural value‐added (constant 2010 USD/ha of agricultural land)**. A higher value‐added indicates a larger more profitable agricultural sector, with widespread use of technology and intensive use of non‐land inputs; thus, suggestive of intensification rather than extensification as key approach to increasing output.	World Bank; OECD; FAO	2017/2016	209	Beach et al., 2015b
	**Ease of doing business (ranked out of 190)**. Measures business regulation, regulatory outcomes, the extent of the legal protection of property, the flexibility of employment regulation, and the tax burden on businesses. Countries that establish a regulatory environment that is conducive to business operations are more likely to mobilize resources from the private sector.	World Bank Doing Business, 2019	2019	178	Ahenkan, 2019; Patel, 2011; Stewart et al., 2009
	**Ease of obtaining a bank loan (indexed 1–7)** with only a good business plan and no collateral. Local actors’ access to credit is key to enable the implementation of new practices across sectors.	World Economic Forum, 2017	2017	136	Bustamante et al., 2014; Madlener et al., 2006
Institutional	Good governance, political stability, and institutional capacity are critical for land‐use actors to implement new practices (z‐scores).				
	**Voice and accountability**	Kaufmann & Kraay, 2019	2019	198	
	**Political stability and absence of violence**			204	Bustamante et al., 2019; da Conceição et al., 2015; Demenois et al., 2020; Djenontin et al., 2018; Doshi & Garschagen, 2020; Regina et al., 2016; Wollenberg et al., 2016
	**Government effectiveness**			202	
	**Regulatory quality**			202	
	**Rule of law**			202	
	**Control of corruption**			203	
	**Tenure insecurity.** Proportion of people who believe it is somewhat or very likely that they could lose the right to use their property or part of it against their will in the next 5 years. Insecure land tenure is a key barrier to investment and the implementation of new practices across sectors.	Global Property Rights Index (Prindex), 2020	2020	135	Descheemaeker et al., 2016; Djenontin et al., 2018; Minang et al., 2014; Robinson et al., 2014; Saito‐Jensen et al., 2015
Geophysical	**Total land‐based technical (biophysical) mitigation potential, by total land area, measured in tCO2/ha**	Our data	2020	212	
Technological	**Access to information and communications** includes access to online governance, media censorship, internet users, and mobile telephone subscriptions. Scored from 0 to 100, limited access to information and communications hinders the ability of local actors to implement updated technological knowledge across sectors.	Social Progress Imperative, 2020	2020	174	Descheemaeker et al., 2016; Grunfeld & Houghton, 2013
	**Market access and infrastructure** measures the quality of the infrastructure that enables trade, and distortions in the market for goods and services. Scored from 0 to 100, lower market access and infrastructure reduces the likelihood of local actors to implement new changes in practice.	(Legatum Institute, 2019)	2019	166	(Descheemaeker et al., 2016; Minang et al., 2014)
	**Agricultural TFP (Total Factor Productivity)**. The output is gross agricultural output (GAO) while input growth is the weighted‐average growth in quality‐adjusted land, labor, machinery power, livestock capital, synthetic NPK fertilizers, and animal feed, where weights are input (factor) cost shares. Countries with a higher TFP on the 66.2 to 222.8 index, indicating more efficient use of land and non‐land inputs, are more likely to prevent further land expansion and effectively implement new practices across sectors.	(USDA, 2019)	Avg. 2014–2016	179	(Villoria, 2019)
Socio‐cultural	Countries with higher social progress levels are more likely to absorb the costs and/or tradeoffs of implementation				(Jewell & Cherp, 2019; Riahi et al., 2017)
	**Personal rights (scored 0–100)** include political rights, freedom of expression, freedom of religion, access to justice, and property rights for women.	(Social Progress Imperative, 2020)	2020	170	
	**Nutrition and basic medical care (scored 0–100)** includes undernourishment, deaths from infectious diseases, child stunting, maternal mortality, and child mortality.			177	
Environmental‐ecological	**EPI (Environmental Performance Index)** assesses environmental health and ecosystem vitality, as well as performance toward environmental targets, using 11 criteria (air quality, sanitation & drinking water, heavy metals, waste management, biodiversity & habitat, ecosystem services, fisheries, climate change, pollution emissions, water resources, and agriculture). Countries with higher scores from 0 to 100 will have higher feasibility of implementing land‐based mitigation measures.	(Wendling et al., 2020)	2020	175	(Djenontin et al., 2018; Dumbrell et al., 2016; Mbow et al., 2014; Mbow et al., 2014; Mbow, Van Noordwijk, et al., 2014; Tvinnereim et al., 2017)

Based on the six IPCC feasibility dimensions (de Coninck et al., 2018).

#### 2.2.3 Harmonization and scaling

Processing of the selected feasibility indicators and associated data was done following a two‐step approach. First, all raw data were scaled from 0 to 100 using the formula: *(x_i_*‐*min(x))*/*(max(x)*‐min(x))*100 where *i* indicates the value of indicator x for a given country. When the raw data were already scaled 0–1, it was then multiplied by 100. Where needed, the data were also harmonized for direction by applying 1−*x*, to ensure that higher feasibility was represented by a higher indicator value as well as to ensure consistency between indicators.

#### 2.2.4 Feasibility score

The final step involved the calculation of feasibility scores by averaging all indicators per category, then averaging each of the six categories. We calculated scores including and excluding autocorrelated indicators (*Score 1 and 2*), then we calculated scores with complete and incomplete country observations (*Score 1a and 1b*). *Score 1 and 2* resulted in very similar feasibility rankings; therefore, we chose to include all indicators. Using all indicators (*Score 1*), we then calculated *Score 1a* by including only countries with complete observations (N=113); and *Score 1b* by including countries with five NAs out of six (N=169). Score 1a and 1b resulted in very similar feasibility scores, although the latter allowed for a larger coverage of countries. As such, Score 1a was chosen as the final country‐specific feasibility score (scores provided in Supplementary Information).

### 2.3 Emissions and drivers

To contextualize regional‐ and country‐level circumstances for adopting and implementing land‐based measures in our results (Section 3.2), we assessed land cover areas (ha), total GHG emissions, land‐based emissions in agriculture and land‐use change, and drivers of agricultural emissions and forest cover loss for each country. There is no current and publicly available data on total emissions per country that combines CO_2_ and non‐CO_2_ emissions from fossil fuels, land‐use change, and agriculture. Therefore, we summed the most recent available data on fossil CO_2_ emissions (5‐year average; 2015–2019) (Crippa et al., 2020), agriculture GHG emissions (5‐year average; 2013–2017) (FAO, 2020a), and land‐use, land‐use change, and forestry (LULUCF) emissions (5‐year average; 2013–2017) (Grassi et al., 2021). For each country, we then calculate cost‐effective mitigation potential as a share of total emissions. To identify the main drivers of land sector emissions in each country, we used agricultural emissions data from FAOSTAT (2020) (5‐year average; 2013–2017), and tree cover loss data from Global Forest Watch (5‐year average; 2013–2017).

## 3 RESULTS

### 3.1 Global

#### 3.1.1 Mitigation potential across land‐based measures

**FIGURE 2:**
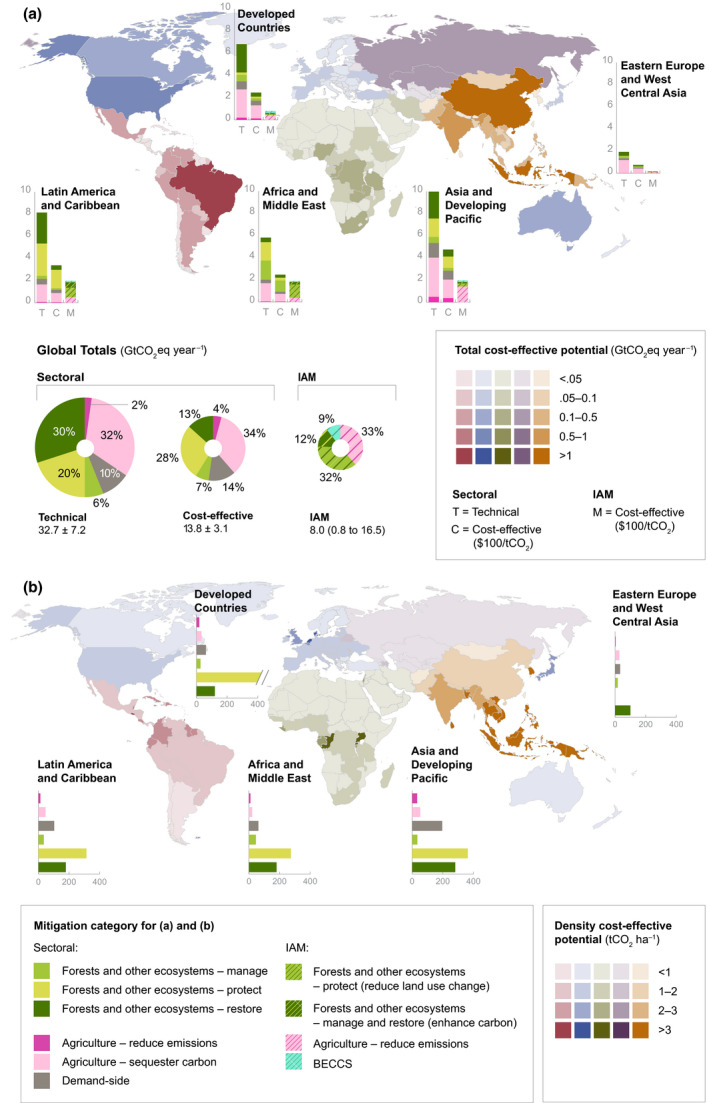
Regional land‐based mitigation potentials. (a) Country‐level map of total cost‐effective ($100/tCO_2_eq) mitigation potential (taking the average potentials for measures with more than one dataset). The five colors on the map correspond to the five IPCC regions assessed in our study. Bar charts show the share of mean technical, cost‐effective, and integrated assessments model (IAM) mitigation by mitigation category, aggregated into the five IPCC regions. Pie charts illustrate global total potentials and share of mitigation potential by mitigation category for sectoral and IAM approaches. Sectoral aggregate potentials exclude BECCS and clean cookstoves to avoid double counting. (b) Country‐level map of cost‐effective mitigation potential density (potential per hectare in 2020–2050). Bar charts show the regional mitigation density by category (cumulative potential divided by total land area per measure per region) for 2020 to 2050. “Protect” measures in Developed Countries show high density due to the very small land area associated with high potential from peatland protection

Between 2020 and 2050, the total cost‐effective mitigation potential (up to $100/tCO_2_eq) of land‐based measures using a sectoral approach is 13.8 ± 3.1 GtCO_2_eq yr^−1^, 42% of the technical potential (Figure 2a). The cost‐effective potential, which represents a more realistic and plausible level of deployment, is a little more than the average annual AFOLU emissions in 2007–2016 of 12 ± 2.9 GtCO_2_eq yr^−1^ (Jia et al., 2019). Using the IAM approach, cost‐effective potential (up to $100/tCO_2_eq) in 2050 is 6.9 median (0.4–11.3 range) for AFOLU (agriculture +land‐use change) and 8.0 median (0.8–16.5 range) for AFOLU +BECCS (total land‐based mitigation) (Figure 2a). The total cost‐effective land‐based mitigation potential from IAMs is 58% of the sectoral potential. The difference is largely due to four main reasons: (1) the IAMs currently incorporate only about a third of land‐based mitigation measures included in the sectoral approach (Figure 1); thus, the inclusion of additional land‐based measures (i.e., wetland protection and restoration, soil carbon sequestration, biochar, agroforestry, and food substitutes) could substantially increase modelled potential; (2) some IAM baselines already have small carbon prices which induce land‐based mitigation, while in others, mitigation, particularly from reduced deforestation is part of the storyline even without an implemented carbon price. Both of these effects dampen the mitigation potential available in the $100/tCO_2_eq carbon price scenario; (3) the IAM estimates include overshoot scenarios which places a substantial portion of mitigation after 2050, especially terrestrial carbon dioxide removal (CDR) options; and (4) it is difficult to completely account for land and resource allocation when aggregating sectoral potentials using different methods, and although we attempt to avoid double counting (see Methods), there is still a risk of overestimation in the aggregate estimates.

**FIGURE 3:**
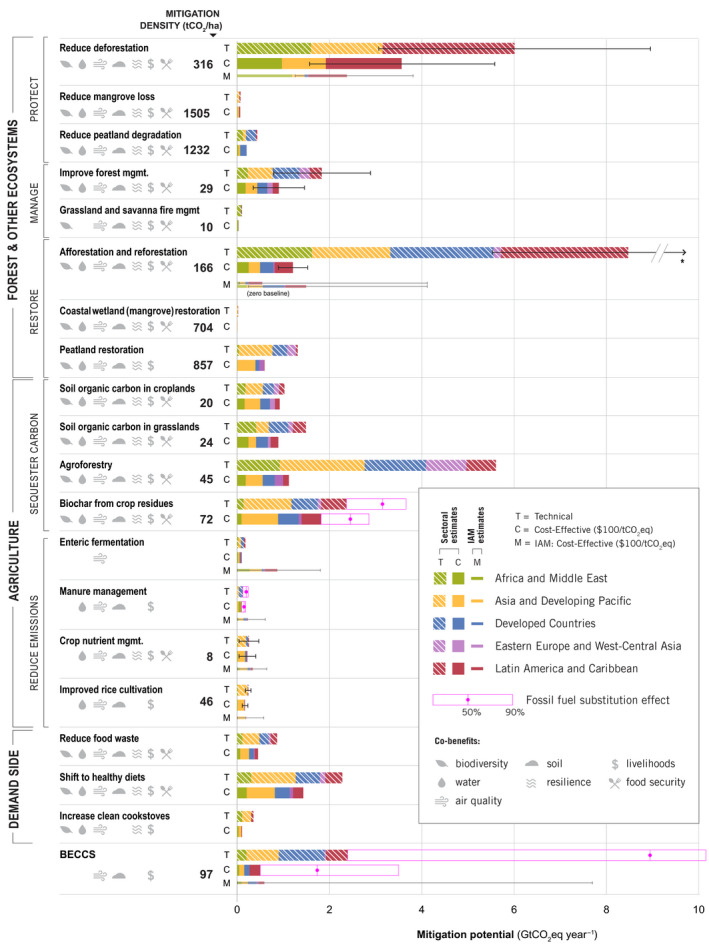
Climate mitigation potentials for 20 land‐based measures in 2020–2050, by region. Technical and cost‐effective ($100/tCO_2_eq) mitigation potentials are provided for each measure using a sectoral approach according to Table 1 and Figure 1. The 20 measures are grouped into four systems‐level mitigation categories, and seven management‐level categories. For measures with more than one dataset, the bar graph represents the mean estimate, and the error bars represent the min and max potential range. Global mitigation potentials of substituting fossil fuels were estimated for BECCS, biochar, and manure management, shown in pink outline bars, illustrating the median and 90^th^ percentile values. IAM estimates (range and median, up to $100/tCO_2_eq) are provided for the seven measures where data are available in the ENGAGE database (Riahi et al., 2021). Potential co‐benefits are indicated with icons, and the average global mitigation “density” (cumulative mitigation potential divided by total hectares in 2020–2050) is noted for measures with available data

Total CDR potential in IAMs, combining land sequestration (A/R, regrowth) and BECCS is 1.7 median (0.2–11.8) GtCO_2_ yr^−1^ up to $100/tCO_2_ in 2050. In the sectoral estimates, CDR potential, which makes up “restore” measures in forests and other ecosystems, and “sequester carbon” measures in agriculture (excluding BECCS to avoid double counting with A/R) is 20.3 ± 3.0 GtCO_2_ yr^−1^ for technical and 6.6 ± 0.3 GtCO_2_ yr^−1^ for cost‐effective (Figure 2a). The sectoral estimates have large CDR potentials from agriculture—agroforestry, biochar, and soil carbon sequestration (4.8 GtCO_2_ yr^−1^ up to $100/tCO_2_)—which are not included in IAMs (Figure 3). The IAM CDR potential is also limited by some A/R deployment in baseline scenarios (see Figure 3 for comparison to a zero baseline) and slower response from A/R in the given timeframe.

Forests and other ecosystems provide the largest share of land‐based mitigation. In sectoral estimates, there is 18.3 ± 6.9 GtCO_2_eq yr^−1^ technical potential and 6.6 ± 2.9 GtCO_2_eq yr^−1^ cost‐effective potential, or 56% and 48% of the total land‐based potential, respectively (Figure 2). In IAMs, cost‐effective potential from land‐use change in forests and other ecosystems in 2050 is 3.5 median (1.4–8.0 range) GtCO_2_eq yr^−1^, 44% of the total land‐based potential. Within forests and other ecosystems in the sectoral estimates, measures that “protect” (reduce deforestation and conversion and degradation of wetlands) make up 20% and 28% of the total technical and cost‐effective potential, respectively, measures that “manage” (improve forest management and grassland fire management) make up 6% and 7%, respectively, while measures that “restore” (A/R, peatland restoration and coastal wetland restoration (mangroves)) make up 30% and 13% (Figure 2a). “Protect” measures make up an increased share of the cost‐effective land‐based mitigation compared to the technical due to its lower cost while “restore” measures decrease by about half due to its higher cost of implementation. Across all land‐based measures, “protect” measures also have the highest mitigation density per year between 2020 and 2050, at an average of about 320 tCO_2_eq ha^−1^, followed by “restore” measures at 175 tCO_2_eq ha^−1^. Protecting mangroves and peatlands have particularly high mitigation densities at about 1500 and 1230 tCO_2_eq ha^−1^ (Figure 3). The protection of primary ecosystems has significant potential for delivering co‐benefits as these ecosystems provide vital ecosystem services (e.g., biodiversity, water and air filtration, livelihoods, food) and can continue to sequester carbon (Figure 3, Supplementary Information). If lost, many natural ecosystems and their carbon stores are also irrecoverable by the 2050 timeframe related to 1.5–2°C pathways and biodiversity goals (Barlow et al., 2007; Goldstein et al., 2020). The potential co‐benefits and possible tradeoffs of measures in forests and other ecosystems depend on how and where the measure is implemented. In the example of A/R, it will depend on the type of species used, scale of deployment (land area is ~1000 Mha to realize technical potential and ~300 Mha for cost‐effective potential), method of deployment (natural regeneration vs mixed species planting vs monoculture planting), and location (ecosystem, climate, and water availability) (Cook‐Patton et al., 2020; Holl & Brancalion, 2020). Tradeoffs from A/R include risks to biodiversity and competition with producing food crops, potentially resulting in indirect land‐use change (Doelman et al., 2020; Kreidenweis et al., 2016).

Agriculture provides the second largest share of land‐based mitigation. The sectoral estimates are 11.3 ± 0.3 GtCO_2_eq yr^−1^ technical potential and 5.3 ± 0.2 GtCO_2_eq yr^−1^ cost‐effective potential, or 34% and 38% of the total land‐based potential, respectively (Figure 2a). In IAMs, cost‐effective potential in agriculture (non‐CO_2_) in 2050 is 2.7 median (0–4.1 range) GtCO_2_eq yr^−1^, 33% of total land‐based potential. IAM estimates for “emissions reductions” in agriculture are over fourfold larger than sectoral estimates (0.6 ± 0.2 GtCO_2_eq yr^−1^ cost‐effective) due to a few factors including higher non‐CO_2_ baseline emissions in the IAMs used, more conservative assumptions of mitigation technology uptake in the sectoral approach, and global economic models used in IAMs capturing additional demand responses and structural changes in agricultural production (Frank et al., 2018). Much of the agriculture potential from sectoral estimates are in “carbon sequestration,” accounting for 32% and 34% of the total technical and cost‐effective potential, respectively. Biochar stands out as the agriculture measure with the highest mitigation density, about 72 tCO_2_eq ha^−1^ between 2020 and 2050, followed by agroforestry and rice cultivation at about 45 tCO_2_eq ha^−1^ each. Biochar also has the potential to mitigate emissions from fossil fuel substitution (Figure 3). The remaining measures have more modest mitigation densities ranging from 8 to 24 tCO_2_eq ha^−1^ as many agriculture measures can be applied across more land (i.e., nutrient management and soil carbon management across a majority of croplands and pasturelands). Unlike measures in forests and other ecosystems (aside from forest management), multiple agriculture measures can often be applied on the same parcel of land. Agriculture measures that enhance soil quality, water efficiency, and yields and reduce pollution—such as soil organic carbon sequestration, agroforestry, biochar, and nutrient management—can provide a relatively wide array of potential co‐benefits (Figure 3, Supplementary Information).

Demand‐side measures provide 3.1 GtCO_2_eq yr^−1^ technical and 1.9 GtCO_2_eq yr^−1^ cost‐effective potential, or 10% and 14% of the total land‐based potential, respectively (Figure 2a). Shifting to sustainable healthy diets makes up 7% and 10% of the total land‐based technical and cost‐effective potential, respectively, and reducing food waste 3% across both potentials. To avoid double counting with reduced deforestation, these sectoral estimates exclude land‐use change impacts from reduced food waste and shifts to healthy diets, as well as clean cookstoves. When the entire value chain is considered (land‐use change emissions and sequestration), the mitigation potential of demand‐side measures increases significantly (+52% for diet shifts and +670% for reduced food waste in our estimates), and have among the highest potentials to mitigate emissions in AFOLU (Bajželj et al., 2014; Roe et al., 2019; Smith et al., 2013; Springmann et al., 2016; Tilman & Clark, 2014). Demand‐side measures are included in IAMs as scenario elements and/or as an endogenous response to food prices, which typically increase in response to carbon prices. Generally, the more sustainable the socioeconomic scenario used, the more diet shifts and food system efficiencies are deployed. Decreasing consumption of high greenhouse gas‐intensive foods like animal‐based proteins, particularly beef, and reducing food loss and waste, reduces land used for feed, water use, and soil degradation, thereby improving efficiency and generating substantial cost savings, increasing resources for improved food security, reducing land competition, and catalyzing and enabling supply‐side measures such as reduced deforestation and reforestation (Figure 3, Supplementary Information).

Estimated mitigation from BECCS is modest, with technical and cost‐effective potential in our sectoral estimate of 2.5 GtCO_2_eq yr^−1^ and 0.5 GtCO_2_eq yr^−1^, respectively (Figure 3). Our estimate only includes the CDR potential, which accounts for the net mitigation, considering the full life‐cycle emissions (land‐use change emissions, forgone sequestration, bioenergy supply chain, etc.). This potential is constrained by the 30‐year payback‐period used here and assuming biomass supply from purpose grown crops only, with potentials increasing at longer evaluation periods or if agricultural or forestry residues were included (Hanssen et al., 2020). BECCS can also provide energy and/or materials which may be used to substitute fossil fuels and could increase mitigation potential by several orders of magnitude (Figure 3). In IAMs, the cost‐effective potential of BECCS is 0.7 (0.01–7.7) GtCO_2_eq yr^−1^ in 2050 (9% of total land‐based potential), just slightly higher than the sectoral estimates. Both sectoral and IAM potentials are lower than previous studies largely due to the $100/tCO_2_eq cost constraint. BECCS potential in IAMs increase substantially with higher carbon prices. In our sectoral estimates, the land area required for BECCS to realize its technical potential is 740 Mha and 160 Mha for cost‐effective potential. Depending on scale and method of deployment, type of biomass supply, and location, BECCS poses tradeoffs and risks for resource use, land competition, and food security. However, if well implemented (e.g., at lower scales and deployed in tandem with forest management, A/R and biochar strategies on marginal or degraded lands), BECCS also has the potential to deliver co‐benefits (Figure 3, Supplementary Information).

#### 3.1.2 Comparing mitigation potential across countries and regions

The top 15 countries with the highest total cost‐effective mitigation potential from land‐based measures are (in descending order) the following: Brazil, China, Indonesia, United States, India, Russian Federation, Canada, the Democratic Republic of the Congo (DRC), Colombia, Mexico, Argentina, Australia, Bolivia, Peru, and Myanmar (Figure 2a). Together, they account for 62% of the global mitigation potential. The countries with highest cost‐effective mitigation potential are generally those with the highest AFOLU emissions. Countries such as Ethiopia and Sudan are an exception, with high AFOLU emissions and relatively lower cost‐effective potential because their emissions are predominantly from livestock, which are costlier to mitigate. Total potential is generally highest in countries with large land areas. However, when the density of mitigation potential (total potential per hectare of land) is considered, some small island states move to the top, largely due to high mitigation potential in protecting or restoring wetlands and forests. The top 15 countries with the highest cost‐effective density potential are (in descending order) as follows: Maldives, Brunei, Bangladesh, Indonesia, Vietnam, Trinidad and Tobago, Malaysia, Malta, Rwanda, South Korea, Netherlands, Cambodia, Mauritius, Philippines, and El Salvador (Figure 2b). The full dataset on mitigation potentials by country is available in the Supplementary Information.

Across the IPCC regions, the highest cost‐effective potentials are found in Asia and developing Pacific with 4.8 ± 1 GtCO_2_eq yr^−1^ (34%), followed by Latin America and Caribbean (3.4 ± 1.2 GtCO_2_eq yr^−1^; 25%), then Africa and Middle East (2.5 ± 0.7 GtCO_2_eq yr^−1^; 18%), Developed countries (2.5 ± 0.1 GtCO_2_eq yr^−1^; 18%), and Eastern Europe and West‐Central Asia (0.8 ± 0.1 GtCO_2_eq yr^−1^; 5%), (Figure 2a). The cost‐effective mitigation potential is 42% of the global technical potential, but with considerable regional variation: 48% is cost‐effective in Asia and developing Pacific, 42% in Africa and Middle East, 41% in Latin America and Caribbean, 36% in Developed countries, and 39% in Eastern Europe and West‐Central Asia. Tropical countries in Asia, Africa, and Latin America have the largest proportions of cost‐effective potential; proportions are lower in developed countries largely due to higher costs of implementation. Additional detail on the five IPCC regions is outlined in Section 3.2 “Five Regions.”

#### 3.1.3 Feasibility across regions and categorization of countries

**FIGURE 4:**
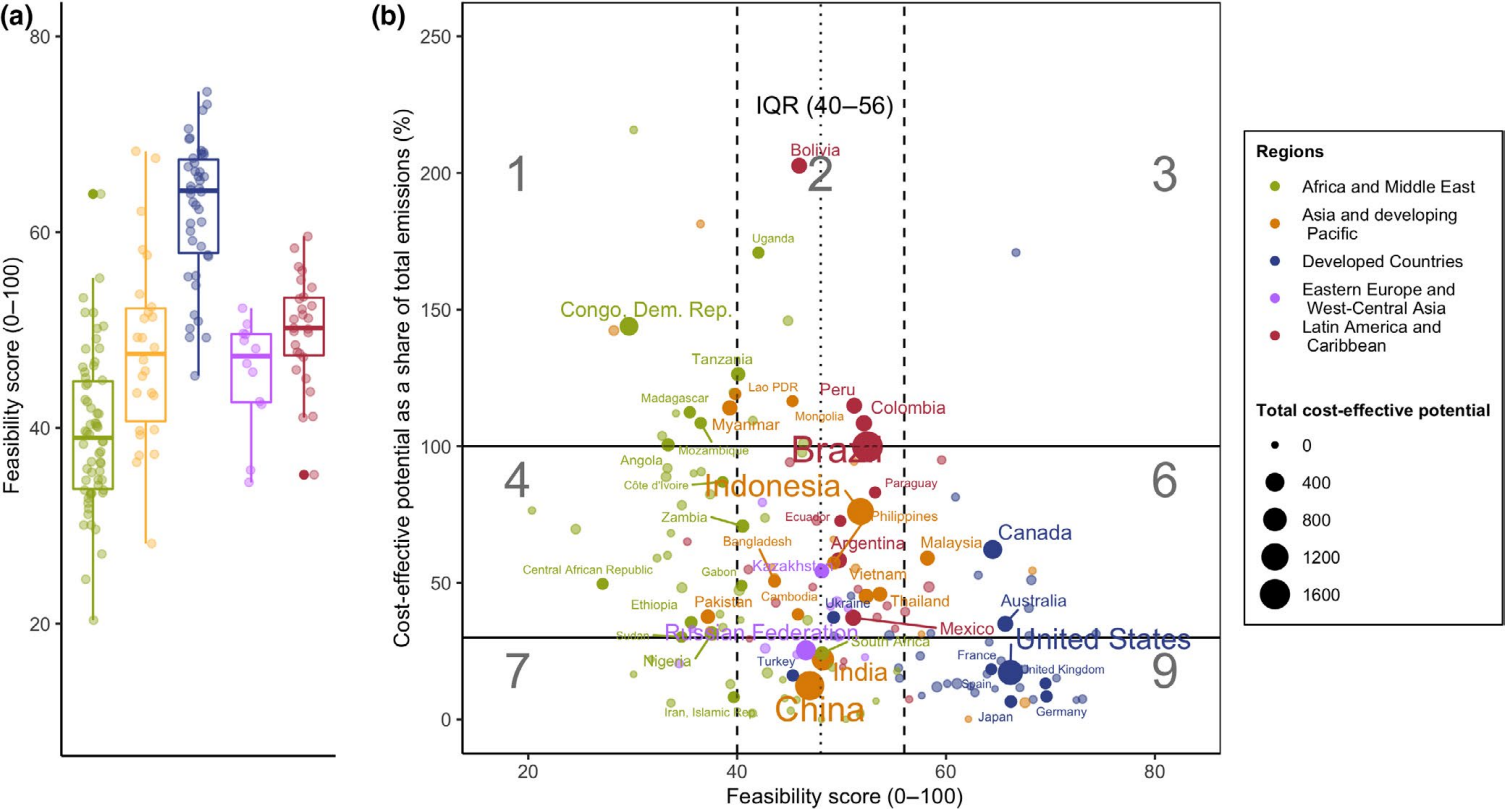
Country feasibility and cost‐effective mitigation potential as a share of total emissions. (a) Boxplot of feasibility scores by region (b) Feasibility score (0–100) by total cost‐effective mitigation potential as percent of total country emissions. Circles show relative size of total cost‐effective potential in GtCO_2_eq yr^−1^. The vertical dashed lines represent the interquartile range and median feasibility scores, and the horizontal lines represent the share of cost‐effective mitigation potential that land‐based measures can deliver over 30% (in line with global 1.5℃ trajectory) and over 100% (can achieve net zero emissions or negative emissions with land‐based measures only). Countries are grouped and numbered into 1–9 categories (except for 5 and 8 to improve data visibility), according to their relative mitigation potential as a share of total emissions and feasibility score. In six countries, the proportion of cost‐effective potential relative to total emissions is higher than the y‐axis of 250%: Papua New Guinea, Republic of Congo, Cameroon, Guyana, Suriname, and Rwanda; these can be seen in Figures 5, 6, 7, 8, 9

Globally, the median feasibility score for implementing land‐based mitigation measures was 48 (40 – 56 IQR), which corresponds approximately to the median scores for developing countries (Figure 4). The highest feasibility scores were for Denmark (74), the Netherlands (73) and Luxembourg (72), while the lowest feasibility scores were for Eritrea (20), Chad (24) and Central African Republic (27). Developed countries had the highest median feasibility scores (64), followed by developing countries (48) and then least developed countries (LDCs) (36). Developed countries had higher scores in five of the six feasibility categories assessed: economic, institutional, technological, social and environmental, while developing countries and LDCs scored higher in the geophysical category. Among developed countries, Denmark (74) was highest overall, among developing countries, Brunei (68) was highest, and among LDCs, Bhutan was highest (51). The Russian Federation was lowest among developed countries, Republic of the Congo among developing countries, and Eritrea, lowest in feasibility among LDCs. Comparisons between regions show that Developed Countries (Europe, North America, Developed Pacific) had a median feasibility score of 64, followed by Latin American and Caribbean countries with 50, Asian and developing Pacific countries with 48, Eastern European and West‐Central Asian countries with 47, and African and Middle Eastern countries with 39.

When feasibility scores are compared to the share of cost‐effective land‐based mitigation potential relative to national emissions, countries can be broadly categorized into nine categories (numbered in Figure 4) of either high, medium or low across the two variables. Countries in the top tier (#1‐3) are those with land‐based mitigation potential greater than 100% of total country emissions, or “Surplus potential” countries. Tropical forest countries with relatively low fossil fuel emissions in Africa, Southeast Asia and Latin America are found in the “Surplus potential” tier, with Iceland as the exception. Countries in the middle tier (#4‐6), or “High relative potential” countries, have land mitigation potentials between 30% and 100% of economy‐wide emission levels, higher than the global average of 20–30% to meet the 1.5°C pathway (Roe et al., 2019). “High relative potential” includes tropical forest countries and large agriculture countries with average fossil fuel emissions. Countries in the lower tier (#7‐9) have lower than 30% of mitigation potential relative to total emissions, largely due to their high levels of fossil fuel emissions and/or low land‐based potential (e.g., desert biomes), thus labelled “Limited relative potential” countries. The feasibility score categories of “low” (<25th percentile), “medium” (25–75 percentile), and “high” (>75th percentile) largely reflect countries’ development level, with LDCs predominantly aggregated in “low”, developing countries in “medium” and developed countries in “high”, with some exceptions including Bhutan (an LDC) with a feasibility score above the 50th percentile and Russia (a developed country) scoring below the 50th percentile. Our characterizations of low, medium, and high feasibility are conceptual and should not be interpreted as sharp distinctions, even though they use numerical thresholds to define different zones.

Of the cost‐effective mitigation potential, 19% is found in countries with “low” feasibility scores, 61% in countries with “medium” feasibility scores, and 20% in countries with “high” feasibility scores. Across feasibility categories, 22% of mitigation potential is located in countries scoring above the global average in 0–1 categories, 58% in 2–4 categories, and 20% in 5–6 categories. These values indicate which categories may be targeted to improve countries’ feasibility scores. For the majority (58%) of countries scoring above global average in 2 to 4 categories, addressing environmental, institutional, and economic barriers would be the most important in unlocking potential (i.e., increasing the feasibility) in this framework.

### 3.2 Five regions

#### 3.2.1 Africa and Middle East

Africa and the Middle East (AME) comprises approximately 35 million km^2^, of which 19% is forest (20.6% primary and 2% planted) and 39% is agricultural land. Total AFOLU emissions were 2.7 GtCO_2_eq yr^−1^ (averaged between 2013 and 2017), 0.9 GtCO_2_eq yr^−1^ (35%) from agriculture and 1.8 GtCO_2_eq yr^−1^ (65%) from land‐use change. The main drivers of agriculture emissions are enteric fermentation (42%), manure left on pastures (30%), and the burning of grasslands and savannahs (17%), whereas the main driver of tree cover loss (proxy for land‐use change) is shifting agriculture (90%), far ahead of commodity production (4%).

**FIGURE 5:**
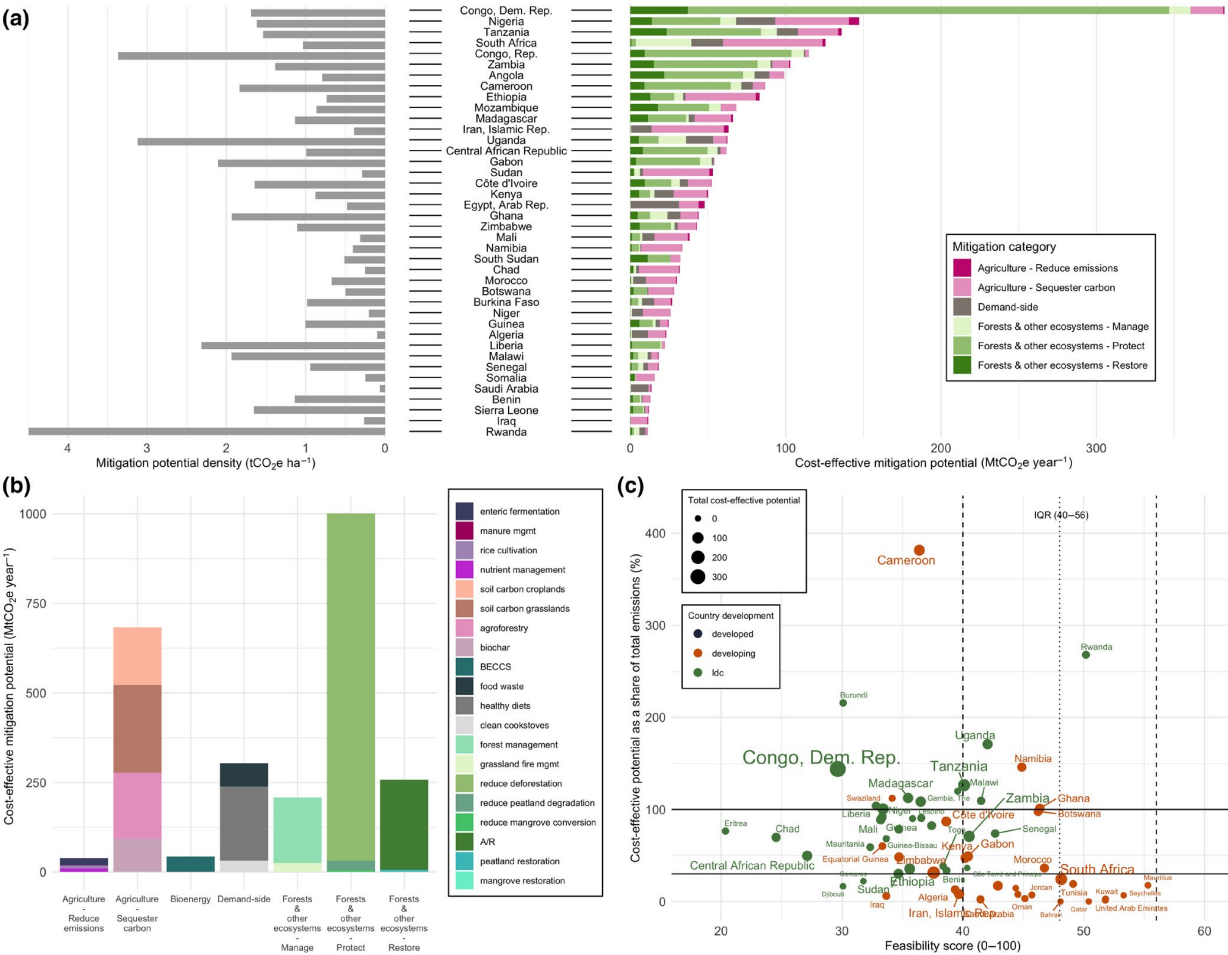
Africa and Middle East (AME) land‐based mitigation potentials and feasibility. (a) Total cost‐effective mitigation potential by mitigation category (colored bars) and mitigation density of cost‐effective potentials (gray bars), by country; (b) Total cost‐effective mitigation potential by mitigation category and measure in AME; c) Feasibility score by cost‐effective mitigation potential as a share of total country GHG emissions (%) in AME

The total technical mitigation potential in AME is 5.8 ± 2.3 GtCO_2_eq yr^−1^, and the cost‐effective mitigation potential ($100/tCO_2_eq) is 2.5 ± 0.7 GtCO_2_eq yr^−1^ (43%). The highest cost‐effective mitigation potential comes from reducing deforestation (0.97 ± 0.4 GtCO_2_eq yr^−1^; 39%), then afforestation and reforestation (0.25 ± 0.2 GtCO_2_eq yr^−1^; 10%), sequestering soil organic carbon in grasslands (0.24 GtCO_2_eq yr^−1^; 10%), shifting diets (0.2 GtCO_2_eq yr^−1^; 8%), and agroforestry (0.19 GtCO_2_eq yr^−1^; 8%) (Figure 5b). The IAM cost‐effective potential (up to $100 per tCO_2_eq) for land‐based mitigation (AFOLU + BECCS) is 1.8 (−0.1–4.8) GtCO_2_eq yr^−1^ in 2050.

Across the countries, the DRC has the most cost‐effective mitigation potential at 0.4 ±0.2 GtCO_2_eq yr^−1^, or about 16% of AME potential (Figure 5a). The DRC is followed by Nigeria, Tanzania, South Africa, Republic of Congo, and Zambia. In the DRC, the Republic of the Congo, Tanzania, and Zambia, where land‐based emissions are largely driven by deforestation from shifting agriculture, “forest protection” measures present the highest cost‐effective mitigation potential. Over half (57%) of AME countries have cost‐effective potentials that are over 30% of their total emissions, or “High relative potential.” In all, 16 countries have cost‐effective potentials exceeding their total emissions, or “Surplus potential” (Figure 5c). Rwanda, Mauritius, the Republic of Congo, and Uganda have the highest mitigation densities at over 3 tCO_2_eq ha^−1^ (Figure 5a). At the regional scale, average mitigation density is at 1 tCO_2_eq ha^−1^, with the protection of forests and other ecosystems offering the highest mitigation density at 274 tCO_2_eq ha^−1^, followed by the restoration of forests and other ecosystems at 180 tCO_2_eq ha^−1^ and improved forest management at 46 tCO_2_eq ha^−1^ (Figure 2b).

The median feasibility score in AME (39) is nine points below the global median, with more than half of AME countries being below the 25th percentile “low” and Israel being the only country above the 75th percentile “high” (Figure 5c). AME countries scored below‐average feasibility compared to global scores in all six feasibility dimensions (economic, institutional, geophysical, technological, socio‐cultural, and environmental‐ecological).

#### 3.2.2 Asia and Developing Pacific

Asia and the developing Pacific (ADP) is approximately 21 million km^2^, of which 28% is forest (22% primary and 20% planted), and 51% is agricultural land. Total AFOLU emissions were 3.3 GtCO_2_eq yr^−1^ (averaged between 2013 and 2017), 2.1 GtCO_2_eq yr^−1^ (63%) from agriculture and 1.2 GtCO_2_eq yr^−1^ (37%) from land‐use change. The main drivers of agriculture emissions are enteric fermentation (32%), rice cultivation (21.5%), and synthetic fertilizers (18%), whereas the main drivers of tree cover loss (proxy for land‐use change) are agricultural commodities (57%) and forestry (27%).

**FIGURE 6:**
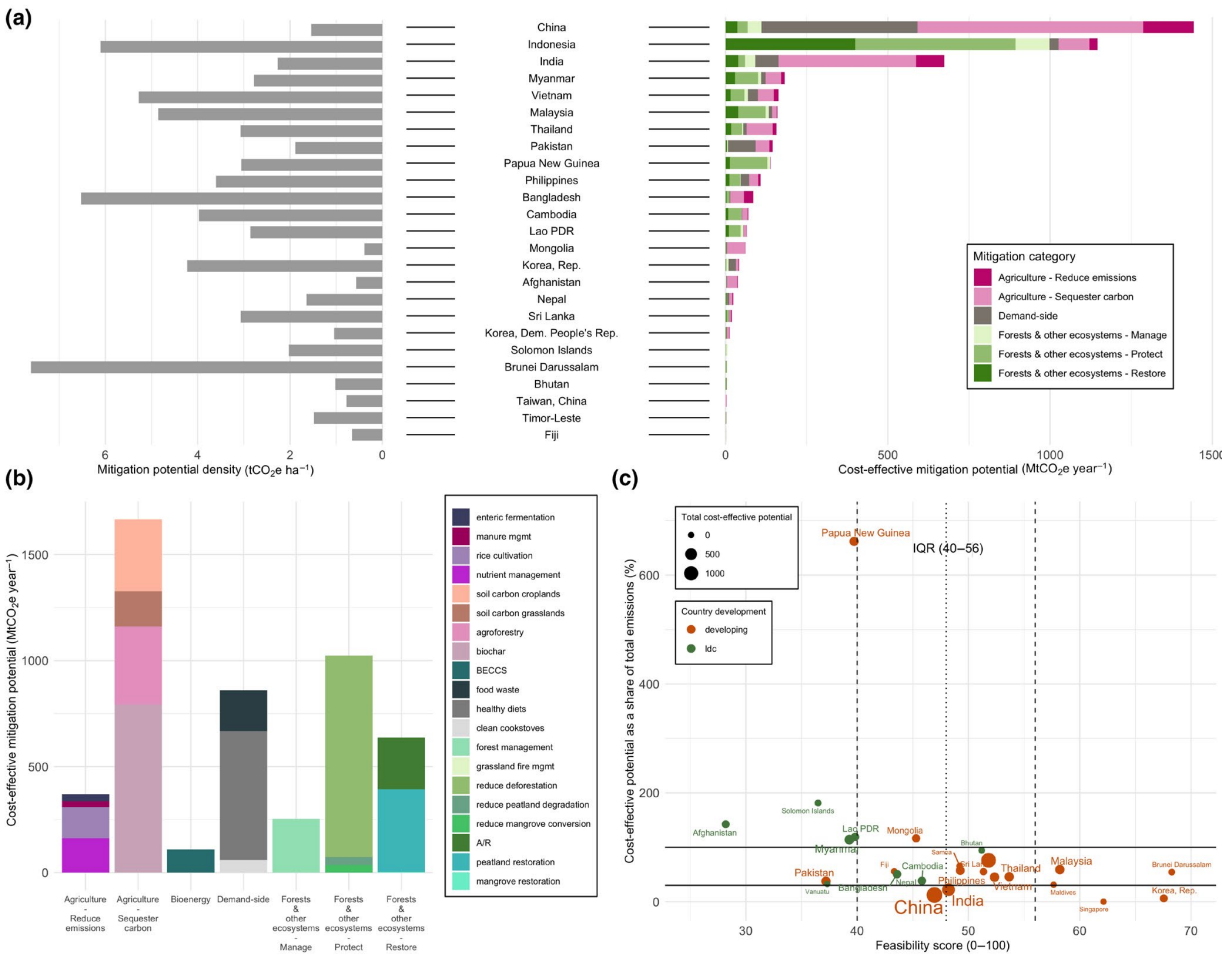
Asia & Developing Pacific (ADP) land‐based mitigation potentials and feasibility. (a) Total cost‐effective mitigation potential by mitigation category (colored bars) and mitigation density of cost‐effective potentials (gray bars), by country; (b) Total cost‐effective mitigation potential by mitigation category and measure in ADP; (c) Feasibility score by cost‐effective mitigation potential as a share of total country GHG emissions (%) in ADP

The total technical mitigation potential in ADP is 10 ± 2.2 GtCO_2_eq yr^−1^, and the cost‐effective mitigation potential ($100/tCO_2_eq) is 4.8 ± 1.0 GtCO_2_eq yr^−1^ (48%). The highest cost‐effective mitigation potential comes from reducing deforestation (0.95 ± 0.6 GtCO_2_eq yr^−1^; 20%), then biochar application (0.8 GtCO_2_eq yr^−1^; 17%), shifting diets (0.6 GtCO_2_eq yr^−1^; 13%), peatland restoration (0.4 GtCO_2_eq yr^−1^; 8%), and agroforestry (0.37 GtCO_2_eq yr^−1^; 8%) (Figure 6b). The IAM cost‐effective potential (up to $100/tCO_2_eq) for land‐based mitigation (AFOLU +BECCS) is 2.2 median (0.8–4.4 range) GtCO_2_eq yr^−1^ in 2050.

Across the countries, China has the highest cost‐effective mitigation potential at 1.4 ± 0.1 GtCO_2_eq yr^−1^, or about 30% in ADP, largely due to its size which is 45% of the land area in ADP (Figure 6a). China is followed by Indonesia, India, Myanmar, and Vietnam (all five countries make up 75% of potential in ADP). China's AFOLU emissions are concentrated in agriculture (97%), accordingly, its largest mitigation potential is from “sequester carbon” measures in agriculture (48%), demand‐side measures (34%), then “reduce emissions” measures in agriculture (11%). Land‐based emissions in Indonesia, Myanmar, and Vietnam are largely driven by deforestation due to commodity production, forestry, and shifting agriculture, and thus have the largest mitigation potential in the protection of forest and other ecosystems. Similar to China, land‐based emissions in India are dominated by enteric fermentation and synthetic fertilizer use, with highest potential from soil carbon sequestration (63% of total potential). Seven countries have “Surplus potential,” or cost‐effective potentials that are over 100% of their total emissions (Figure 6c). About half (49%) of ADP countries have cost‐effective potentials that are over 30% of their total emissions (“High relative potential” tier). The Maldives, Brunei, Bangladesh, Indonesia, and Vietnam have the highest mitigation densities at over 5 tCO_2_eq ha^−1^ (Figure 6a), although the first two countries have relatively modest total potentials due to their small size. At the regional scale, mitigation density is 2.3 tCO_2_eq ha^−1^, with the protection (363 tCO_2_eq ha^−1^) and restoration (281 tCO_2_eq ha^−1^) of forests and other ecosystems offering the highest mitigation density, followed by “sequester carbon” measures in agriculture (53 tCO_2_eq ha^−1^) (Figure 2b).

Countries in ADP are evenly distributed on either side of the global median with regards to their feasibility scores, with most countries being located in the 50th–75th percentiles, “medium.” Brunei, the Republic of Korea, Malaysia, the Maldives, and Singapore are above the 75% percentile, “high,” while Afghanistan, Lao PDR, Myanmar, Pakistan, Papua New Guinea, the Solomon Islands, and Vanuatu are below the 25% percentile “low” (Figure 6c). Relative to global scores, ADP countries scored below‐average in five feasibility dimensions (economic, institutional, technological, socio‐cultural, and environmental‐ecological) and above‐average scores in the geophysical dimension.

#### 3.2.3 Developed countries

Developed countries (DC) cover approximately 33 million km^2^, of which 31% is forest (32% primary and 12% planted), and 37% is agricultural land. Total AFOLU emissions were 1.25 GtCO_2_eq yr^−1^ (averaged between 2013 and 2017), 1.1 GtCO_2_eq yr^−1^ (87%) from agriculture and 0.17 GtCO_2_eq yr^−1^ (13%) from land‐use change. The main drivers of agriculture emissions are enteric fermentation (37%), synthetic fertilizer use (18%), and manure deposition on pasture (12%), whereas the main driver of tree‐cover loss is forestry (76%).

**FIGURE 7:**
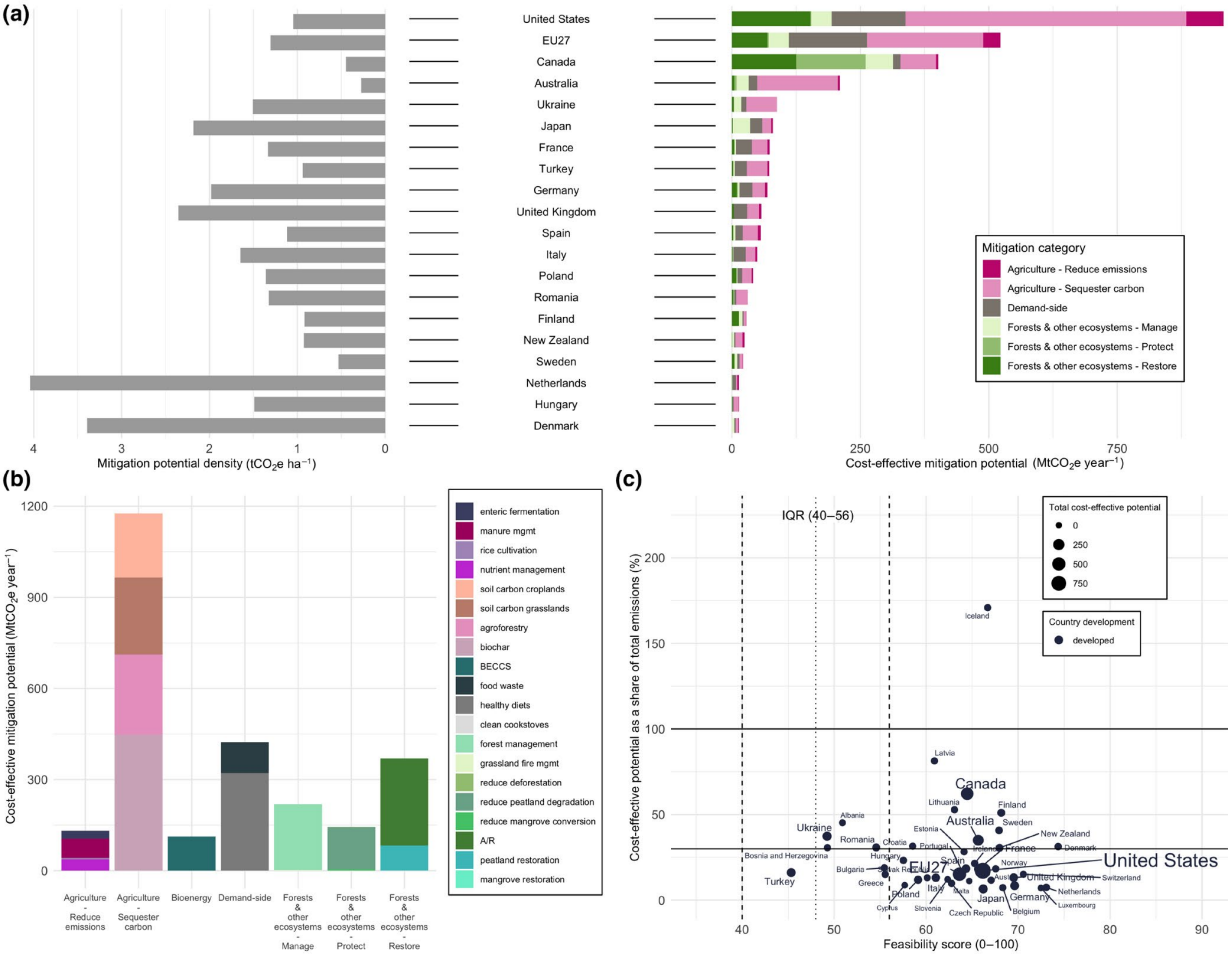
Developed countries (DC) land‐based mitigation potentials and feasibility. (a) Total cost‐effective mitigation potential by mitigation category (colored bars) and mitigation density of cost‐effective potentials (gray bars), by country. EU27 represents the 27 European Union countries as of 2021; (b) Total cost‐effective mitigation potential by mitigation category and measure in DC; (c) Feasibility score by cost‐effective mitigation potential as a share of total country GHG emissions (%) in DC

The total technical mitigation potential in DC is 6.8 ± 0.3 GtCO_2_eq yr^−1^, and the cost‐effective mitigation potential ($100/tCO_2_eq) is 2.5 ± 0.1 GtCO_2_eq yr^−1^ (36%). The IAM cost‐effective potential (up to $100 per tCO_2_eq) for land‐based mitigation (AFOLU + BECCS) is 1.0 median (−0.1–3.0 range) GtCO_2_eq yr^−1^ in 2050. The highest cost‐effective mitigation potential comes from biochar application (0.45 GtCO_2_eq yr^−1^; 18%), shifting to healthy diets (0.32 GtCO_2_eq yr^−1^; 13%), afforestation and reforestation (0.29 ± 0.04 GtCO_2_eq yr^−1^; 12%), agroforestry (0.26 GtCO_2_eq yr^−1^; 11%), soil organic carbon sequestration in grasslands (0.25 GtCO_2_eq yr^−1^; 10%), and improved forest management (0.22 ± 0.1 GtCO_2_eq yr^−1^; 9%) (Figure 7b).

Across the countries in DC, the United States (US) has by far the largest cost‐effective mitigation potential at 0.96 ± 0.05 GtCO_2_eq yr^−1^, 39% of the potential (Figure 7a), followed by Canada (0.40 ± 0.02 GtCO_2_eq yr^−1^, 16%), Australia (0.21 ± 0.02 GtCO_2_eq yr^−1^, 9%), Ukraine (0.09 ± 0.01 GtCO_2_eq yr^−1^, 4%), and Japan (0.08 ±0.02 GtCO_2_eq yr^−1^, 3%). When the EU27 (current European Union countries) is aggregated, it has the second highest mitigation potential at 0.52 ± 0.04 GtCO_2_eq yr^−1^, 21%. The land‐based emissions from the top countries are primarily driven by agriculture, as such, the highest cost‐effective mitigation potentials are in “sequester carbon” measures (highest proportion of the US’, EU27’s Australia's and Ukraine's total cost‐effective potentials), followed by demand‐side measures. Forest measures (protection, reforestation, and forest management) also provide significant potentials across these countries, representing the highest opportunities for Canada and Japan, respectively. In all, 14 DC countries have cost‐effective potentials that are over 30% of their total emissions, “High relative potential,” while Iceland is the only country to have cost‐effective potential exceeding its total emissions “Surplus potential” (Figure 7c). Bermuda, Malta, the Netherlands, and Denmark have the highest mitigation densities, more than 3 tCO_2_eq ha^−1^ (Figure 7a). At the regional scale, average mitigation density is 0.74 tCO_2_eq ha^−1^, with the protection of forests and other ecosystems offering the highest mitigation density at about 1150 tCO_2_eq ha^−1^ (very high as the estimate mostly covers a small area of peatlands and coastal wetlands) followed by the restoration of forests and other ecosystems at 120 tCO_2_eq ha^−1^ and “sequester carbon” measures in agriculture at 32 tCO_2_eq ha^−1^ (Figure 2b).

The median feasibility score in DC (62.3) is well above the global median, a vast majority of DC countries being above the 75th percentile, or “high” feasibility (Figure 7c). For the remaining countries, eight are in the 50th–75th percentiles, Turkey is the only country in the 25%–50% percentiles, and no DC country scored under the 25th percentile. DC countries obtained above‐average scores compared to global scores in five out of the six feasibility dimensions (all but the geophysical dimension).

#### 3.2.4 Eastern Europe and West‐Central Asia

Eastern Europe and West‐Central Asia (EEWA) is approximately 21 million km^2^, of which 41% is forest (33% primary and 3% planted) and 25% is dedicated to agriculture. Total AFOLU emissions were 0.2 GtCO_2_eq yr^−1^ (averaged between 2013 and 2017), 0.19 GtCO_2_eq yr^−1^ (95%) from Agriculture and 0.01 GtCO_2_eq yr^−1^ (5%) from land‐use change. The main drivers of agriculture emissions are enteric fermentation (46%), manure management (11%), and synthetic fertilizers (10%), whereas the main drivers of tree cover loss are wildfires (59%) and forestry (35%).

**FIGURE 8:**
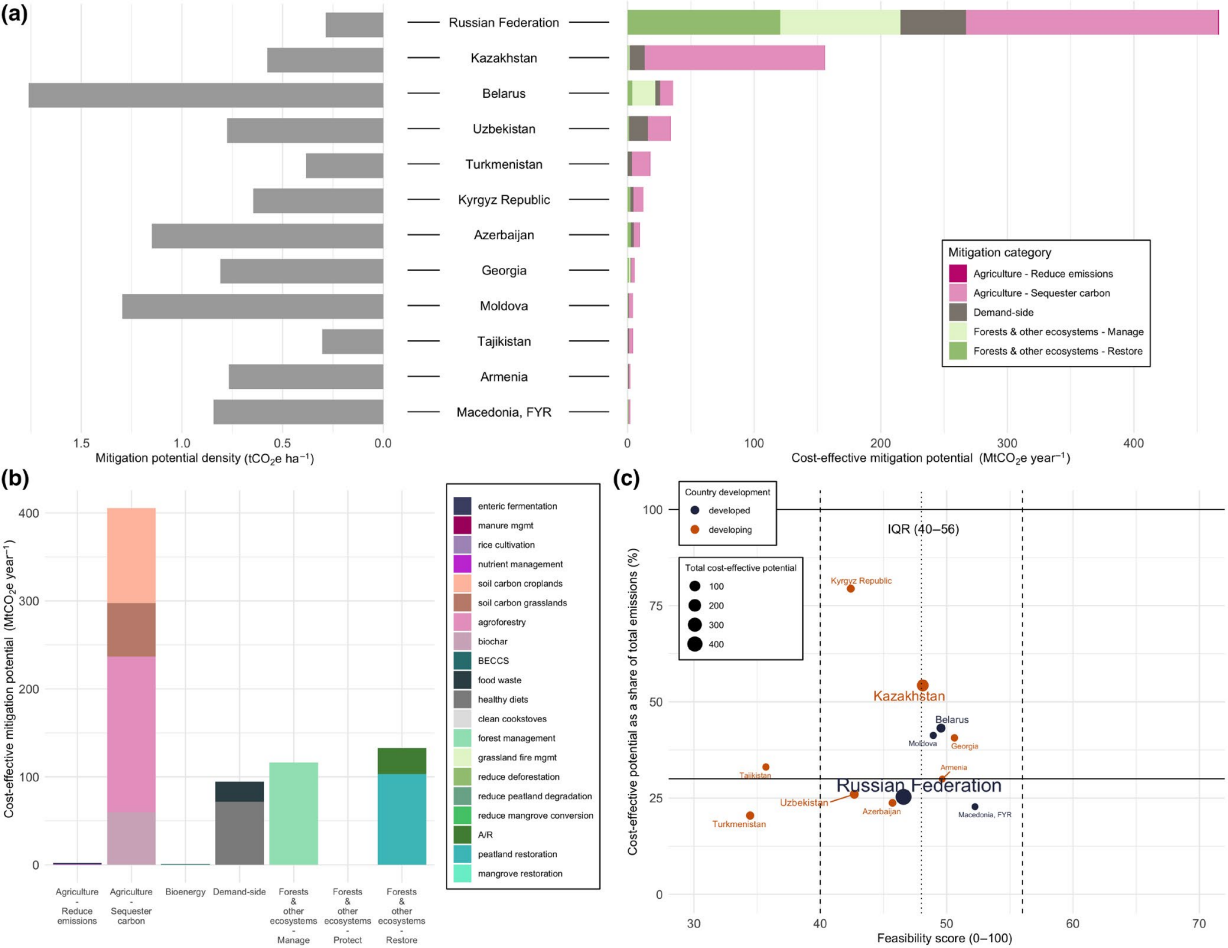
Eastern Europe and West‐Central Asia (EEWA) land‐based mitigation potentials and feasibility. (a) Total cost‐effective mitigation potential by mitigation category (colored bars) and mitigation density of cost‐effective potentials (gray bars), by country; (b) Total cost‐effective mitigation potential by mitigation category and measure in EEWA; (c) Feasibility score by cost‐effective mitigation potential as a share of total country GHG emissions (%) in EEWA

The total technical mitigation potential in EEWA is 1.9 ± 0.1 GtCO_2_eq yr^−1^, and the cost‐effective mitigation potential ($100/tCO_2_eq) is 0.75 ± 0.1 GtCO_2_eq yr^−1^ (39%). The highest cost‐effective mitigation potential comes from agroforestry (0.18 GtCO_2_eq yr^−1^; 24%), then forest management (0.12 ± 0.08 GtCO_2_eq yr^−1^; 16%), soil organic carbon in croplands (0.11 GtCO_2_eq yr^−1^; 13%), peatland restoration (0.1 GtCO_2_eq yr^−1^; 13%), and shifting diets (0.07 GtCO_2_eq yr^−1^; 10%) (Figure 8b). The IAM cost‐effective potential (up to $100/tCO_2_eq) for land‐based mitigation (AFOLU + BECCS) is 0.12 (0.04–0.7) GtCO_2_eq yr^−1^ in 2050.

Across the countries, Russia has the largest cost‐effective mitigation potential at 0.47 ± 0.05 GtCO_2_eq yr^−1^, or about 62% in EEWA, largely due to its size which is 78% of the land area in EEWA (Figure 8a). The Russian Federation is followed by Kazakhstan, Belarus, Uzbekistan, and Turkmenistan. The land‐based emissions in these countries are attributed to agriculture, and the highest cost‐effective mitigation potentials are in agricultural carbon sequestration measures (except for Belarus, where improved forest management measures have the highest potentials due to the importance of their forestry sector on emissions). Demand‐side measures are also important in Russia, Kazakhstan, and Uzbekistan. Six EEWA countries have cost‐effective potentials that are over 30% of their total emissions, “High relative potential,” however, unlike in other regions, none have cost‐effective potential exceeding their total emissions (Figure 8c). Belarus has the highest mitigation density, at 1.8 tCO_2_eq ha^−1^. At the regional scale, average mitigation density is fairly low, 0.36 tCO_2_eq ha^−1^, with the restoration of forests and other ecosystems offering the most mitigation density at about 100 tCO_2_eq ha^−1^, followed by carbon sequestration in agriculture at 28 tCO_2_eq ha^−1^ (Figure 2b).

The median feasibility score in EEWA (47) is slightly below the global median, with half of EEWA countries in the 50th–75th percentiles and one‐third in the 25–50th percentiles (all “medium” feasibility). No EEWA country lies in the 75%–100% percentiles, while Tajikistan and Turkmenistan are below the 25% percentile, or “low” feasibility (Figure 8c). EEWA countries have below‐average scores in five feasibility dimensions (institutional, geophysical, technological, environmental‐ecological, and socio‐cultural), and above‐average scores in the economic dimension.

#### 3.2.5 Latin America and Caribbean

Latin America and the Caribbean (LAC) is approximately 20 million km^2^, of which 47% is forest (46% primary and 3% planted) and 36% is dedicated to agriculture. Total AFOLU emissions were 2.2 GtCO_2_eq yr^−1^ (averaged between 2013 and 2017), 0.9 GtCO_2_eq yr^−1^ (40%) from agriculture and 1.3 GtCO_2_eq yr^−1^ (60%) from land‐use change. The main drivers of agriculture emissions are from livestock production, enteric fermentation (58%), and manure left on pasture (23%), whereas the main drivers of tree cover loss (proxy for land‐use change) are commodity agriculture (51%) and shifting agriculture (38%).

**FIGURE 9:**
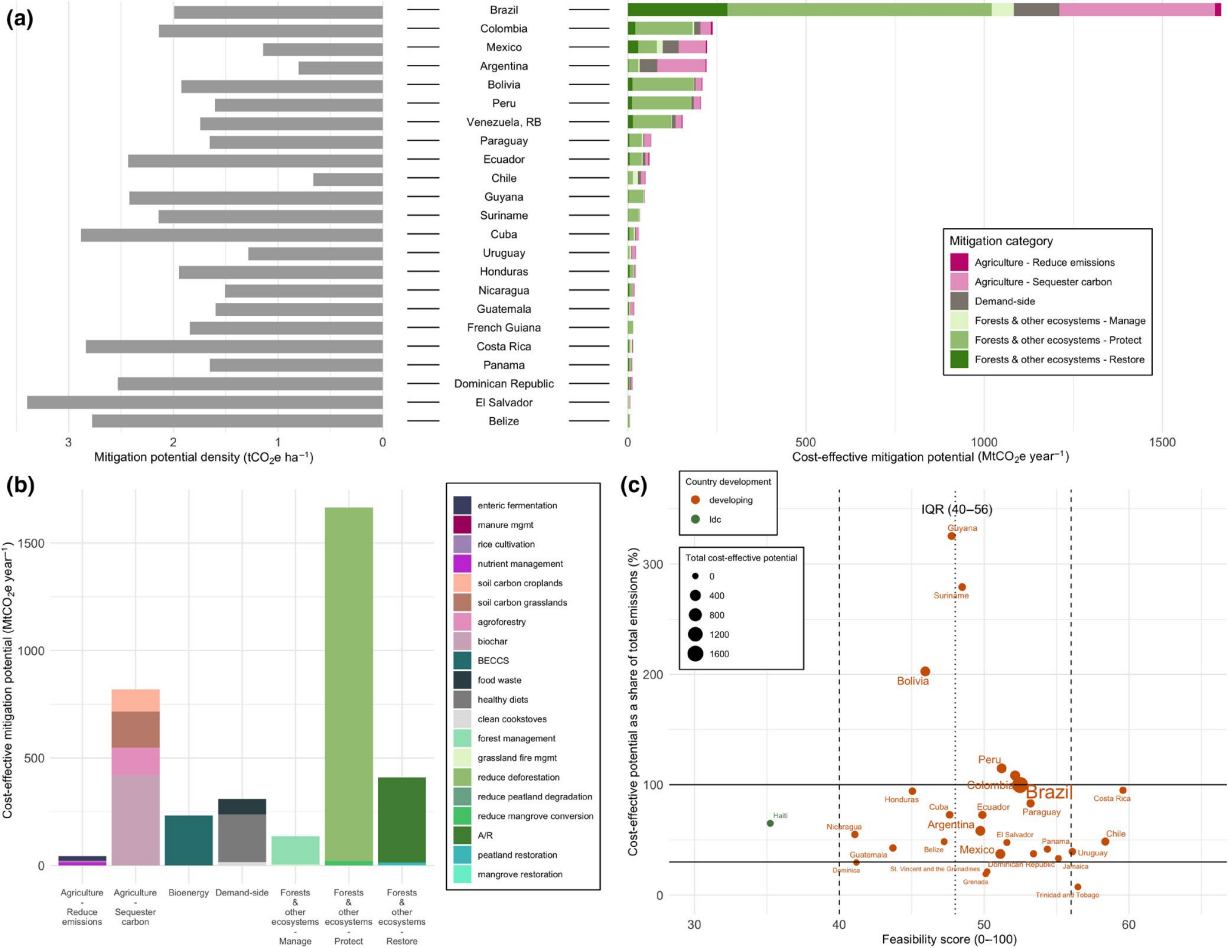
Latin America & Caribbean (LAC) land‐based mitigation potentials and feasibility. (a) Total cost‐effective mitigation potential by mitigation category (colored bars) and mitigation density of cost‐effective potentials (gray bars), by country; (b) Total cost‐effective mitigation potential by mitigation category and measure in LAC; (c) Feasibility score by cost‐effective mitigation potential as a share of total country GHG emissions (%) in LAC

The total technical mitigation potential in LAC is 8.1 ± 2.3 GtCO_2_eq yr^−1^, and the cost‐effective mitigation potential ($100/tCO2eq) is 3.4 ± 1.2 GtCO_2_eq yr^−1^ (42%). The highest cost‐effective mitigation potential comes from reducing deforestation (1.6 ± 0.96 GtCO_2_eq yr^−1^; 49%), then biochar application (0.42 GtCO_2_eq yr^−1^; 13%), A/R (0.4 ± 0.1GtCO_2_eq yr^−1^; 12%), BECCS (0.23 GtCO_2_eq yr^−1^; 7%), shifting diets (0.22 GtCO_2_eq yr^−1^; 7%), soil organic carbon in grasslands (0.17 GtCO_2_eq yr^−1^; 5%), and agroforestry (0.13 GtCO_2_eq yr^−1^; 4%) (Figure 9b). The IAM cost‐effective potential (up to $100 per tCO_2_eq) for land‐based mitigation (AFOLU + BECCS) is 1.9 (0.2–3.8) GtCO_2_eq yr^−1^ in 2050.

Among the LAC countries, Brazil has the highest cost‐effective mitigation potential by several orders of magnitude at 1.7 ± 0.5 GtCO_2_eq yr^−1^, accounting for about 50% in LAC, largely due to its size which is 42% of the land area in LAC (Figure 9a). Brazil is followed by Colombia, Mexico, Argentina, and Bolivia which are predominantly high forest and/or high meat‐producing and consuming countries and thus have protecting forests, restoring forests, shifting to healthy diets, and carbon sequestration in agriculture among the highest potentials (Figure 9b). A large majority (>70%) of LAC countries have cost‐effective potentials that are over 30% of their total emissions, higher than the global median to achieve a 1.5°C trajectory, or “High relative potential.” High forest and lower fossil fuel emissions countries, Guyana, Suriname, Bolivia, Peru, Colombia, Brazil, and Costa Rica all have cost‐effective potentials that are over 100% of their total emissions, or “Surplus potential” (Figure 9c). The density of cost‐effective mitigation potentials (total potential by total area) across all countries is 1.7 tCO_2_eq ha^−1^ (Figure 9a). Trinidad and Tobago, El Salvador, and Barbados have the highest mitigation densities, at >3 tCO_2_eq ha^−1^, even though they have relatively modest total potentials compared to the other countries in the region (Figure 9a).

Most countries in LAC have higher feasibility scores than the global median and are in the 50–75% percentiles (“medium” feasibility). Costa Rica, Chile, Trinidad and Tobago, and Uruguay are above the 75% percentile (“high” feasibility), while Haiti is below the 25% percentile (“low feasibility”) (Figure 9c). Relative to global scores, LAC countries scored below‐average in four feasibility dimensions (economic, institutional, geophysical, and environmental‐ecological) and above‐average scores in the technological and socio‐cultural dimensions.

## 4 DISCUSSION AND CONCLUSIONS

In this study, we provide a comprehensive and updated assessment of global, regional, and country‐level land‐based mitigation potential, and examine country‐level feasibility. We show that our sectoral portfolio of 20 land‐based mitigation activities has the potential to deliver 13.8 ± 3.1 GtCO_2_eq yr^−1^ within the cost‐effective range (up to $100/tCO_2_eq), about 40% of the technical potential. The land‐based mitigation potential across the integrated assessment models (IAMs) is 8.0 median (0.8–16.5 range) within the cost‐effective range (up to $100/tCO_2_eq), about 60% of the sectoral estimate. Combining both approaches, we conclude that the likely range of cost‐effective land‐based mitigation is 8–13.8 GtCO_2_eq yr^−1^ between 2020 and 2050. Cost‐effective mitigation potentials represent a more realistic and actionable target grounded in public willingness to pay for climate mitigation, and therefore, are more relevant in policy‐making than technical potentials.

Our land‐based mitigation estimates are broadly in line with previous studies including the IPCC‐AR5 AFOLU economic mitigation potential of 7.2–10.6 GtCO_2_eq yr^−1^ in 2030 (Smith et al., 2014); the UNEP Emissions Gap potential of 12 (9–15 uncertainty range) GtCO_2_eq yr^−1^ in 2030 (6.7 and 5.3 GtCO_2_eq yr^−1^ for agriculture and forests, respectively) (UNEP, 2017); the cost‐effective potential of 11 GtCO_2_eq yr^−1^ in 2030 estimated by Griscom et al. (2017); and the median supply‐side potential (including technical and economic) of 10.6 GtCO_2_eq yr^−1^ and 1.5ºC land sector roadmap of 14–15 GtCO_2_eq yr^−1^ between 2030 and 2050 from Roe et al. (2019). Our sectoral estimate of 13.8 ± 3.1 GtCO_2_eq yr^−1^ is also on par with modelled 1.5°C pathways for the land sector, 13.8 median (9.9–17.6 IQR) GtCO_2_eq yr^−1^ in 2050 (Roe et al., 2019). Our work builds on and includes several advances (detailed in Methods 2.1) beyond these and other previous studies on land‐based mitigation (Griscom et al., 2017, 2020; Jia et al., 2019; Roe et al., 2019; Smith et al., 2013, 2014; UNEP, 2017), including the first cost‐effective potential for demand‐side measures and soil organic carbon sequestration in croplands and grasslands (full dataset in Supplementary Information). Compared to previous studies, our estimates present lower BECCS potential due to the $100/tCO_2_eq cost constraint, lower demand‐side potential as it does not include emissions reductions from land‐use change to avoid double counting, and higher biochar and soil carbon management potential due to refined methods that capture a broader set of activities.

Our land‐based cost‐effective potential is roughly 50% from forests and other ecosystems, 35% from agriculture and 15% from demand‐side measures (Figure 2a). When the full value chain emissions of demand‐side measures are considered, their potential increases threefold. Each of the 20 land‐based measures incorporated in our study has potential co‐benefits and risks, depending on how and where they are implemented (Figure 3, Supplementary Information). Protection of forests and other ecosystems, particularly of primary ecosystems, and demand‐side measures present high mitigation efficiency, high provision of co‐benefits, and relatively lower costs. However, feasibility barriers, including economic, institutional, and technological constraints (Figure 4), could limit countries from realizing their climate mitigation potentials and the associated co‐benefits. A substantial portion of the global cost‐effective potential (80%) is in developing countries and LDCs, where feasibility issues are of greatest concern.

### 4.1 Data advances made, but gaps remain

Despite the advances made in this study, certain limitations and gaps remain. As previously outlined in the Methods, completely accounting for land competition, and avoiding double counting of mitigation, is difficult when aggregating sectoral estimates from different activities and methodologies. Separate studies may allocate the same land for divergent abatement activities. We attempt to limit double counting by excluding certain measures that could overlap (Methods 2.1.1). While we can limit overlapping activities, we are not able to adequately account for land competition and suboptimal allocation of land and feedstocks when combining all activities from our sectoral approach assessed in Table 1. Due to these limitations, we also provide a comparison with IAM estimates that account for land allocation and optimization across all economic sectors, and thus avoid double counting. IAMs, however, have other limitations. As outlined in the Methods Section 2.1.2 and Results 2.1.1, 3.1.1, IAMs only include about one‐third of the land‐based measures in the sectoral estimates, and thus may be underestimating mitigation potential in the land sector. In addition to a more limited portfolio of land‐based measures, IAMs are generally coarser in resolution than sectoral studies, and a majority do not provide country‐level estimates. IAM mitigation potential estimates also have large ranges as different models and scenarios vary in their baseline assumptions (e.g., some already include carbon prices and reduced deforestation in baseline which reduce mitigation potential) and timing of mitigation (e.g., some scenarios generate temperature overshoots which place most of the mitigation after 2050—beyond the time horizon considered in our estimates).

Our estimates (both sectoral and IAMs), as with most current land‐based mitigation estimates, do not account for (1) substitution effects for avoiding fossil fuel emissions (although we provide global estimates for BECCS, biochar, and manure management); (2) foregone sequestration potential from avoided land‐use change (with the exception of the BECCS estimate); and (3) potential impacts from future climate change. These issues could have a substantial impact on land‐based mitigation globally and regionally. Substitution effects of land‐based measures, particularly of BECCS, biochar and wood products have the potential to reduce significant fossil fuel emissions. Accounting for the continued carbon sequestration potential of protecting forests and other ecosystems, rather than just avoided emissions, would also increase mitigation potential. On the other hand, inadequate action to reduce atmospheric GHG concentrations enhances the risk that climate impacts will reduce future potential for land‐based mitigation and turn residual land sinks into sources (Jia et al., 2019). Additional research is therefore needed on the impact of substitution effects, foregone sequestration, and climate change impacts on individual land‐based mitigation activities at a regional or country level. More data on country‐level tradeoffs (e.g., biodiversity impacts, resource‐use limitations) from land‐based measures could also aid country‐level planning. Finally, expanding the portfolio of land‐based mitigation measures in IAMs (e.g., non‐forest ecosystems, soil carbon sequestration in agriculture, demand‐side measures) and country‐level sectoral approaches (e.g., blue carbon from seagrass and marshes, savanna and grassland restoration, management of hard wood products, enhanced rock weathering) would broaden the range of AFOLU potential considered.

### 4.2 Global and temporal implications of land‐based mitigation

To stay on a 1.5°C pathway, total emissions will need to fall by about 50% each decade, until net zero emissions are reached about mid‐century (Rockström et al., 2017; Roe et al., 2019; Rogelj et al., 2018). This process will require the transformation of every economic sector (Rogelj et al., 2018). Because of their economic characteristics, their substantial co‐benefits, their ability to work in tandem with the decarbonization of other sectors, and their potential for rapid implementation, land‐based mitigation activities could provide a large share of the near‐term (next decade), low‐cost mitigation necessary to meet such ambitious decadal milestones. Although some land‐based mitigation potentials could be realized comparatively quickly, current levels of financing and investments in land‐based mitigation and nature‐based solutions (UNEP, 2021) continues to be inadequate in unlocking mitigation at the cost‐effective levels outlined in our study. Mobilizing sufficient investments in the next few years will be critical for near‐term mitigation gains. Longer‐term opportunities which require more time to realize mitigation gains, like carbon sequestration measures (A/R, soil carbon management) and/or additional research, technology and development, such as the deployment of BECCS, will need up‐front investment and long‐term land‐use planning including risk mitigation.

Our analysis adds new dimensions relevant to strategic planning and successful implementation of land‐based measures, which can be used to plan and prioritize country‐specific policies and measures that target co‐benefits and help achieve other international goals and targets, such as the goals formulated under the NYDF and the UN Decade on Ecosystem Restoration, and the SDGs. Land‐based mitigation potential roughly correlates with countries’ land area, but our analysis of mitigation densities reveals that many smaller countries have disproportionately high levels of mitigation potential for their size, suggesting fertile ground for targeted investments. Our feasibility assessment also suggests that weak governance, low economic development, limited access to technology, socio‐cultural conditions, and low acceptance of policies could create barriers for implementing land‐based mitigation, particularly in developing countries and LDCs. However, implementation barriers as well as opportunities also depend on the type and site‐specific location of land‐based measure. Collaborative efforts to reduce barriers and open opportunities at the country or regional level may release globally significant quantities of near‐term mitigation at relatively low costs. The timing, quantity, co‐benefits, and cost are key considerations for external actors who seek to help these countries mobilize their mitigation potential. Our research suggests that investments to increase feasibility and readiness may prove to be more cost‐effective than investments aimed at the land‐based mitigation activities themselves (i.e., by helping shift countries from left to right in Figures 4, 5, 6, 7, 8, 9, mobilizing mitigation that might otherwise be infeasible).

### 4.3 Country context for implementing and scaling‐up action

Our results show that the opportunities among countries are quite heterogeneous, in terms of the relative scale of mitigation potential, the types of land‐based measures available, their potential co‐benefits and risks, and the feasibility of realizing them. Strategies that determine what, where, when, and how mitigation measures are implemented will therefore vary significantly by country. Implementing mitigation measures that maximize co‐benefits and limit risks will require strategies that consider mitigation costs and opportunities in other sectors, environmental and socio‐economic consequences across stakeholders, consider tradeoffs and spillovers among mitigation actions and with other policy goals, and budgetary implications. To aid the development of such strategies, it is helpful to look at individual country plans and glean lessons learned from experiences in implementing land‐based mitigation measures and policies. We highlight three countries below according to three mitigation potential tiers “Limited relative potential,” “High relative potential,” and “Surplus potential” (Figure 4, Section 3.1.3) to outline some lessons and considerations in scaling‐up action.

China, a “Limited relative potential, medium feasibility” country, recently announced a long‐term climate mitigation plan to peak emissions before 2030 and achieve net zero emissions, or carbon neutrality, by 2060. To achieve its goals, China has to restructure its economy (Mallapaty, 2020), including a 90% reduction of all GHG emissions by 2050 compared to 2005 levels and carbon removals using natural carbon sinks such as A/R and other CDR technologies (Tianjie, 2020). China has significant experience with large‐scale A/R programs, including the Grain for Green initiative to mitigate soil erosion, that resulted in a 25% net increase in global canopy area on 6.6% of global vegetated area between 2000 and 2017 (Chen et al., 2019). However, some of China's afforestation efforts generated significant localized tradeoffs such as water depletion and reduced biodiversity, which led to criticisms of, and adjustments to government programs (Hua et al., 2016). China's long‐term climate mitigation plan highlights the need to harmonize climate with sustainable development goals. However, China has not yet included policy targets or measures for maintaining healthy diets or reducing food waste, which make up about 35% of its cost‐effective land‐based mitigation potential and can deliver significant co‐benefits. China is an example of an industrialized country which, as a matter of priority, has to decarbonize its energy and industrial sectors (>90% of its emissions), but can use AFOLU mitigation to tap into near‐term mitigation potentials that can deliver social and environmental co‐benefits. Furthermore, any efforts to shift diets and reduce food waste could alter the long‐term trajectory of agriculture emissions in China and beyond, especially considering its role as a major importer of agricultural commodities, including those that cause deforestation.

In contrast, the Democratic Republic of Congo, a “Surplus potential, low feasibility country,” is characterized by relatively low fossil fuel emissions and high AFOLU emissions. DRC has the potential to generate surplus AFOLU mitigation, largely through the protection of forests and other ecosystems (95%), that can enable the country to achieve net negative emissions by mid‐century. However, according to their NDC, the DRC faces a series of feasibility challenges that undermine the deployment and scaling up of mitigation action: limited national financial resources, external financial support, and technical, jurisdictional and institutional capacity; as well as the absence of policies and incentives that adequately addresses competing sectoral interests (mining, agriculture and forestry) (Government of the Democratic Republic of the Congo, 2015). Activating DRC’s mitigation potential will require addressing drivers of deforestation (commercial agriculture (40%), subsistence farming (20%), or wood fuel harvesting (20%) and development challenges at the nexus of food security, rural development, energy supply, and forest conservation. Various programs and initiatives to reduce deforestation in the DRC have been in place since 2015 (Central African Forest Initiative created, FCPF Readiness Package approved); however, funding has been slow to materialize and feasibility constraints make it difficult for DRC to access result‐based finance. DRC is an example of a forest LDC country that would significantly benefit from deploying an integrated development strategy that leapfrogs carbon‐intensive development in favor of clean and sustainable development choices, and from international partnership and assistance.

Another example, Ecuador, is a “High relative potential, medium feasibility country” with large potentials for protecting forests and other ecosystems (~60%). Reducing deforestation is identified as one of the main mitigation options in the country's NDC, which proposes to reduce deforestation by 4% (unconditional) or 20% (conditional on support) compared to a 2000–2008 reference level (Government of the Republic of Ecuador, 2015). The country's existing payment‐for‐ecosystem services program, established in 2008 (Acuerdo Ministerial 161, Plan Nacional del Buen Vivir), proves the ability to successfully realize AFOLU mitigation potentials while delivering substantial co‐benefits including ecosystem services and income to forest communities. Landowner contracts are for 20 years and commit to the preservation of tree cover. As of December 2018, almost 175,000 people participated in the program, resulting in estimated avoided deforestation of 1.6 Mha, spanning about 15% of Ecuador's territory (Ecuadorian Ministry of Environment, 2018). The program also led to a decrease in land conflicts in areas with ambiguous land titles (Jones et al., 2020) and generated both socioeconomic and ecological benefits. However, the program depends on continued government funding to incentivize persistent conservation behavior (Etchart et al., 2020). Ecuador expanded its funding sources for conservation programs by receiving results‐based finance from the REDD+ Early Movers program (Germany/Norway, signed 2018) and the Green Climate Fund (2019). The country's experience with payment‐for‐ecosystem services shows how conservation payments can strengthen land governance but also that continued funding and support is essential for its success.

These country examples within our country categories (Figure 4, Section 3.1.3) highlight various important considerations in implementing and scaling‐up land‐based mitigation. (1) AFOLU mitigation strategies are more successful when part of long‐term strategies and policies that have a holistic view of emissions and decarbonization options from other sectors, of various land‐use needs and challenges, and of sustainable economic development (Hurlbert et al., 2019). (2) Allowing for adaptive adjustments over time could enable needed corrections and enhance program sustainability and effectiveness (Hurlbert et al., 2019; Smith et al., 2020). (3) The integration of global commodity markets means that demand‐side measures should complement local supply‐side measures. Embedded emissions and carbon leakage, particularly for large agricultural importers, make it difficult for medium‐ or low‐feasibility countries to collectively address AFOLU emissions, particularly where agricultural demand and economic opportunity act as drivers of deforestation (Pendrill et al., 2019). While demand‐side measures are largely lacking in country NDCs, they are essential to achieve AFOLU potentials. (4) Developing and LDC countries will need to continue to develop, and could benefit from leap‐frogging fossil‐fuel intensive infrastructure and moving directly to sustainable energy infrastructure (Levin & Thomas, 2016). (5) Global cooperation and tailored assistance could help address feasibility barriers in developing countries, particularly to increase economic and institutional capacity and to help develop country‐specific plans to start implementation.

## Supporting information

Supplementary Material
